# Development of 2-Aminotetralin-Type Serotonin 5-HT_1_ Agonists: Molecular Determinants for Selective Binding and Signaling at 5-HT_1A_, 5-HT_1B_, 5-HT_1D_, and 5-HT_1F_ Receptors

**DOI:** 10.1021/acschemneuro.3c00658

**Published:** 2023-12-27

**Authors:** Ryan P. McGlynn, Meng Cui, Brittany Brems, Otto Holbrook, Raymond G. Booth

**Affiliations:** † Center for Drug Discovery, Northeastern University, Boston, Massachusetts 02115, United States; ‡ Department of Pharmaceutical Sciences, Northeastern University, Boston, Massachusetts 02115, United States; § Department of Chemistry and Chemical Biology, Northeastern University, Boston, Massachusetts 02115, United States

**Keywords:** aminotetralin, molecular modeling, serotonin receptors, structure activity relationships

## Abstract

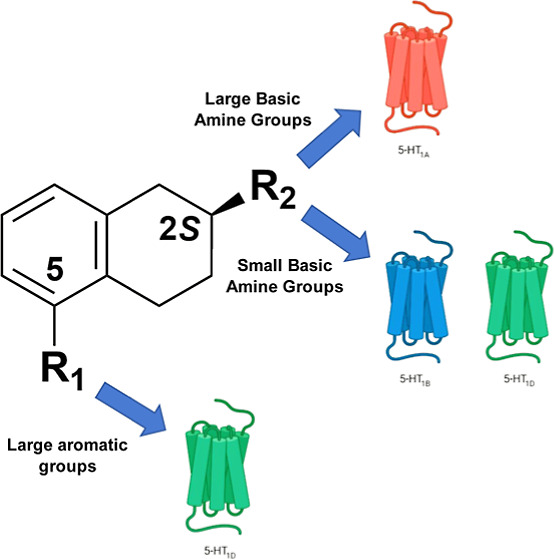

The serotonin (5-hydroxytryptamine, 5-HT) 5-HT_1_ G-protein coupled receptor subtypes (5-HT_1A/1B/1D/1E/1F_) share a high sequence homology, confounding development of subtype-specific ligands. This study used a 5-HT_1_ structure-based ligand design approach to develop subtype-selective ligands using a 5-substituted-2-aminotetralin (5-SAT) chemotype, leveraging results from pharmacological, molecular modeling, and mutagenesis studies to delineate molecular determinants for 5-SAT binding and function at 5-HT_1_ subtypes. 5-SATs demonstrated high affinity (*K*_i_ ≤ 25 nM) and at least 50-fold stereoselective preference ([2*S*] > [2*R*]) at 5-HT_1A_, 5-HT_1B,_ and 5-HT_1D_ receptors but essentially nil affinity (*K*_i_ > 1 μM) at 5-HT_1F_ receptors. The 5-SATs tested were agonists with varying degrees of potency and efficacy, depending on chemotype substitution and 5-HT_1_ receptor subtype. Models were built from the 5-HT_1A_ (cryo-EM), 5-HT_1B_ (crystal), and 5-HT_1D_ (cryo-EM) structures, and 5-SATs underwent docking studies with up to 1 μs molecular dynamics simulations. 5-SAT interactions observed at positions 3.33, 5.38, 5.42, 5.43, and 7.39 of 5-HT_1_ subtypes were confirmed with point mutation experiments. Additional 5-SATs were designed and synthesized to exploit experimental and computational results, yielding a new full efficacy 5-HT_1A_ agonist with 100-fold selectivity over 5-HT_1B/1D_ receptors. The results presented lay the foundation for the development of additional 5-HT_1_ subtype selective ligands for drug discovery purposes.

## Introduction

The physiological functions of serotonin (5-hydroxytryptamine, 5-HT) are mediated through 6 families of G-protein coupled receptors (GPCRs) (5-HT_1_, 5-HT_2_, 5-HT_4_, 5-HT_5_, 5-HT_6_, 5-HT_7_)^1^ as well as the 5-HT_3_ cys-loop cation channel family^2^ and the serotonin neurotransporter.^3^ 5-HT_1_-type GPCRs represent the largest family of 5-HT receptors, consisting of the 5-HT_1A_, 5-HT_1B_, 5-HT_1D_, 5-HT_1E_, and 5-HT_1F_ receptor subtypes.^4^ 5-HT_1_-type receptors have high amino acid sequence homology,^5^ high affinity for 5-HT,^6^ and their canonical signaling function is mediated through activation of Gα_i/o_ proteins that subsequently inhibit adenylyl cyclase activity and cyclic adenosine monophosphate (cAMP) formation.^7^ Within the family, 5-HT_1A_, 5-HT_1B_, and 5-HT_1D_ receptors display high affinity for synthetic agonist 5-carboxamidotryptamine (5-CT),^8−10^ a property not seen at 5-HT_1E_ and 5-HT_1F_ receptors.^11^

The 5-HT_1A_ subtype is widely and highly expressed pre- and postsynaptically in many brain regions including the hippocampus,^12^ substantia nigra,^13,14^ and nucleus accumbens.^15^ The 5-HT_1B_ subtype also is highly and widely expressed in the brain, especially in the basal ganglia and frontal cortex.^16^ Unlike 5-HT_1A_ receptors, there is a high concentration of 5-HT_1B_ receptors found in the peripheral nervous system, mainly in the arteries of the cardiovascular system,^17^ which causes concern about cardiotoxicity of 5-HT_1B_-activating drugs.^18^ Compared to 5-HT_1A_ and 5-HT_1B_ receptors, the 5-HT_1D_ receptor has lower brain expression, confined mainly to the basal ganglia, hippocampus, and raphe.^19^ The human 5-HT_1E_ receptor is mainly expressed in brain cortical regions such as the putamen, amygdala, and caudate; unfortunately, there is no apparent rodent homologue, confounding progress in delineating its role in human physiology.^20^ The 5-HT_1F_ receptor is widely expressed in the brain including the dorsal raphe nucleus, hippocampus, cerebral cortex, striatum, thalamus, and hypothalamus, and the receptor plays a critical role in regulating cerebral neurogenic inflammation.^21^

**Figure 1:**
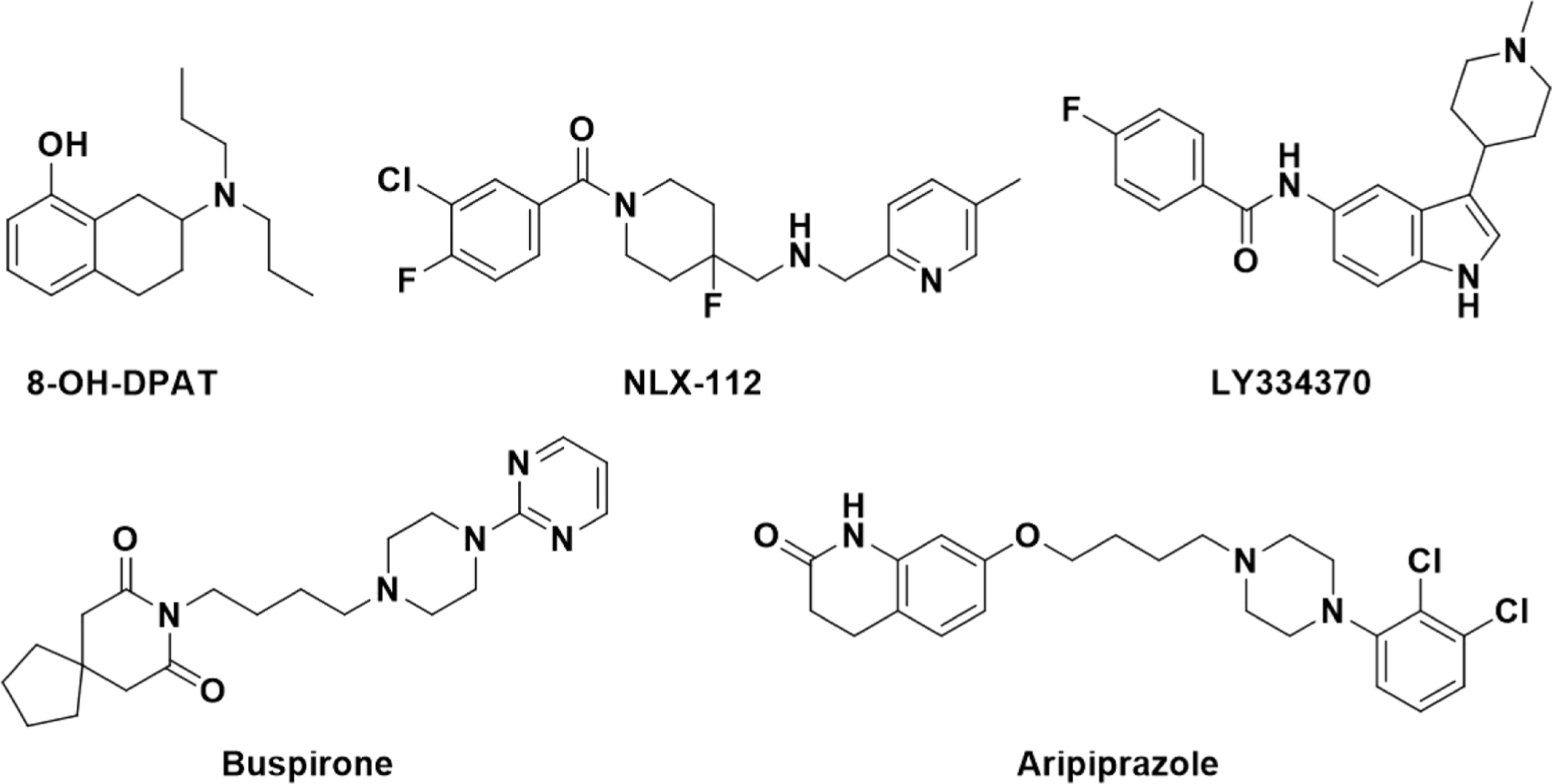
Structures of reference 5-HT_1_ receptor agonists.

Due to widespread expression of 5-HT_1_ receptor subtypes, modulation of their signaling activity suggests important pharmacotherapeutic possibilities, especially for brain disorders. For example, buspirone (Figure 1) is a 5-HT_1A_ receptor partial agonist long approved^22^ to treat generalized anxiety disorder,^23^ and its close congener gepirone recently was approved to treat major depressive disorder.^24^ The activity of buspirone at other 5-HT_1_ receptor subtypes does not appear to be reported, but buspirone is known to have off target liabilities at 5-HT_2A_,5-HT_2B_, 5-HT_2C_, D_2_, D_3_, D_4_, α_1_, and α_2A_ GPCRs.^25^ Similarly, aripiprazole (Figure 1), a potent and high efficacy agonist at 5-HT_1A_ receptors (low functional potency at other 5-HT_1_-subtypes^26^), has prominent procognitive and prosocial clinical activities and is approved to treat schizophrenia, bipolar disorder, and depression.^27,28^ However, aripiprazole also acts at several other neurotransmitter receptor systems.^29^ Recently, NLX-112 (Figure 1), a highly selective 5-HT_1A_ receptor full agonist, entered a phase II clinical trial for l-dopa-induced dyskinesia in Parkinson’s disease.^30^ Sumatriptan, a relatively selective agonist for 5-HT_1B_ and 5-HT_1D_ receptors, is approved for treatment of acute migraines;^31^ however, there are concerns about cardiotoxicity associated with Sumatriptan and other triptans due to cardiovascular expression of 5-HT_1B_ receptors.^32,33^ Lasmiditan selectively activates 5-HT_1F_ receptors and was the first “ditan” approved to treat acute migraine headache,^34^ without the potential for adverse side-effects seen with 5-HT_1B_ and 5-HT_1D_ receptor-active triptans.^35^ Additionally, 5-HT_1A_ receptor agonism has been shown to alleviate tonic nociceptive^36^ as well as neuropathic pain.^37^ In summary, the 5-HT_1_ receptor subtypes represent promising targets for drug development for various disorders.

While there are no approved drugs that selectively target individual 5-HT_1A_, 5-HT_1B_, or 5-HT_1D_ receptor subtypes, there are several commercially available pharmaceutical-like compounds that selectively activate 5-HT_1A_ receptors. For example, 8-hydroxy-*N*,*N*-dipropyl-1,2,3,4-tetrahydronaphthalen-2-amine (8-OH-DPAT, Tables 1 and 2) and NLX-101^38^ are selective 5-HT_1A_ agonist ligands used in research; however, there are no reports about the molecular determinants for their high selectivity at 5-HT_1A_ receptors to guide selective ligand/drug design. Additionally, LY-334370, an analogue of lasmiditan, is a highly selective 5-HT_1F_ agonist^39^ used in research.

**Table 1:**
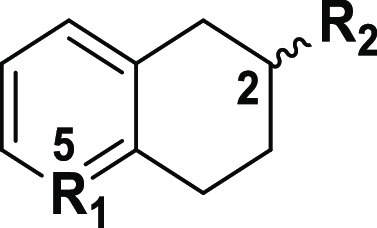

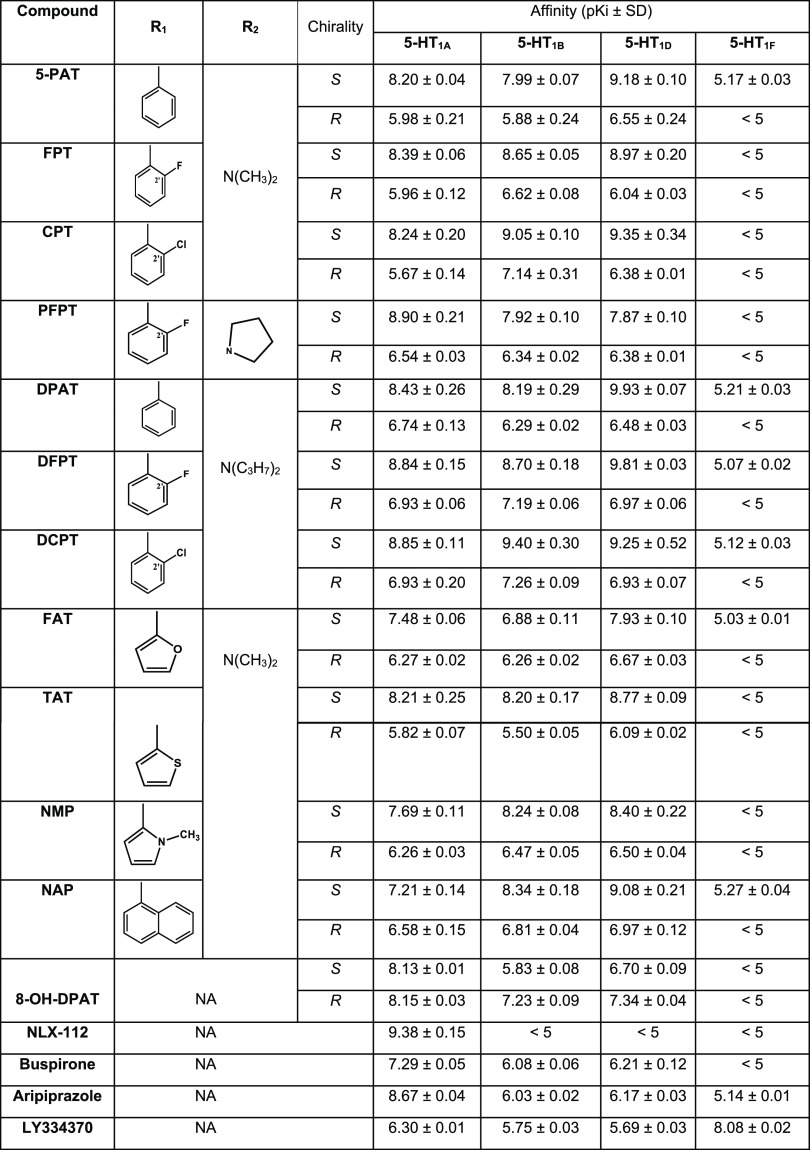
Binding Affinities of 5-SATs and Reference Ligands at 5-HT_1A_, 5-HT_1B_, 5-HT_1D_, and 5-HT _1F_ Receptors^a^

a NA: not applicable—(structures in Figure 1). Dissociation constants (*K*_D_) of [^3^H]5-CT at 5-HT_1A_ = 2.5 nM ± 0.2, 5-HT_1B_ = 5.35 nM ± 0.5, 5-HT_1D_ = 0.82 nM ± 0.1. *K*_D_ of [^3^H]5-HT at 5-HT_1F_ = 7.8 nM ± 2.3.

**Table 2:** Effects of 5-SATs and Reference Ligands on cAMP Accumulation in HEK_t_ Cells Expressing 5-HT_1_ Receptor Subtypes^a^

	**5-HT1A**		**5-HT1B**		**5-HT1D**	
compound	pEC_50_	*E*_MAX_ (%)	pEC_50_	*E*_MAX_ (%)	pEC_50_	*E*_MAX_ (%)
**5-PAT**	7.15 ± 0.30	100 ± 2	7.84 ± 0.29	28 ± 3	9.13 ± 0.32	97 ± 2
**FPT**	7.35 ± 0.12	90 ± 2	9.31 ± 0.08	100 ± 2	8.64 ± 0.61	103 ± 3
**CPT**	7.36 ± 0.15	91 ± 2	9.46 ± 0.29	100 ± 1	8.65 ± 0.07	101 ± 1
**PFPT**	8.78 ± 0.04	100 ± 2	7.25 ± 0.01	99 ± 2	7.24 ± 0.05	98 ± 3
**DPAT**	7.51 ± 0.21	100 ± 1	7.10 ± 0.08	18 ± 3	8.87 ± 0.36	103 ± 2
**DFPT**	7.90 ± 0.10	100 ± 2	8.91 ± 0.17	100 ± 2	8.93 ± 0.12	99 ± 2
**DCPT**	7.58 ± 0.29	92 ± 4	9.54 ± 0.16	103 ± 2	8.62 ± 0.20	101 ± 2
**NAP**	6.41 ± 0.03	72 ± 4	7.46 ± 0.07	30 ± 4	8.06 ± 0.22	90 ± 3
**FAT**	6.31 ± 0.06	92 ± 4	6.93 ± 0.06	102 ± 3	7.92 ± 0.15	100 ± 2
**TAT**	7.63 ± 0.07	91 ± 3	7.80 ± 0.16	85 ± 4	7.38 ± 0.12	60 ± 3
**NMP**	7.56 ± 0.19	70 ± 5	7.95 ± 0.08	54 ± 5	8.08 ± 0.05	66 ± 6
**(S) 8-OH-DPAT**	8.16 ± 0.05	70 ± 3	5.51 ± 0.29	85 ± 3	6.20 ± 0.15	94 ± 2
**(R) 8-OH-DPAT**	8.31 ± 0.33	95 ± 4	6.96 ± 0.16	87 ± 4	7.43 ± 0.17	96 ± 2
**buspirone**	7.34 ± 0.04	75 ± 1	<5	45 ± 3	<5	50 ± 3
**NLX-112**	7.49 ± 0.05	100 ± 3	NR	NR	NR	NR
**aripiprazole**	8.22 ± 0.12	96 ± 2	5.27 ± 0.07	99 ± 3	5.28 ± 0.08	96 ± 1
**5-CT**	8.42 ± 0.23	100 ± 2	8.46 ± 0.18	100 ± 1	9.27 ± 0.24	101 ± 1

a NR: no response at 10 μM.

Due to high sequence homology within the 5-HT_1_ receptor family (at least 60% across subtypes), development of subtype-selective ligands is a challenge. The 5-HT_1B_ and 5-HT_1D_ receptors share the highest sequence homology (95%),^40^ and, unsurprisingly, triptans have high affinity at both receptors. Lack of 5-HT_1B_ and 5-HT_1D_ receptor selective ligands confounds the possible pharmacotherapeutic role(s) of targeting individual receptors. However, recently solved active state cryo-EM structures of the 5-HT_1A_ and 5-HT_1D_ receptors,^41^ along with previous reports of the active state 5-HT_1B_ receptor crystal structure,^42,43^ show intriguing binding pocket differences between subtypes. We exploited recent structural information regarding the 5-HT_1A_, 5-HT_1B_, and 5-HT_1D_ receptors here to employ a structure-based approach for selective ligand design, focusing on our novel 5-substituted-2-aminotetralin (5-SAT) chemotype.

Our lead 5-SAT **FPT** (Table 1) is an agonist at 5-HT_1A_, 5-HT_1B_, and 5-HT_1D_ receptors^44^ that reduced repetitive behaviors and spontaneous seizures as well as increased prosocial behaviors in a mouse model of fragile X syndrome,^45^ the most common monogenetic form of autism spectrum disorder; moreover, **FPT** was orally active and did not cause sedation.^46^ The contribution of each 5-HT_1_ receptor subtype regarding **FPT** preclinical neurotherapeutic activities is unknown, however, and **FPT** has other receptor activities.^47−49^

Herein, we expanded our molecular pharmacology and medicinal chemistry program to characterize activities of 25 5-SAT analogues at 5-HT_1_ subtypes, with the goal to develop subtype-selective ligands. Thus, we report the affinity and function structure–activity relationship (SAR) of 5-SAT analogues at 5-HT_1A_, 5-HT_1B_, 5-HT_1D_, and 5-HT_1F_ receptors. In addition, molecular modeling and molecular dynamics (MD) simulations as well as mutagenesis studies were used to contextualize 5-SAT SAR and validate molecular determinants for selective activation of 5-HT_1_-subtypes, leading to the design and synthesis of new subtype selective 5-SATs.

## Results and Discussion

### Binding Affinities of 5-SATs and Reference Ligands at 5-HT_1_-Type Receptors

Syntheses of 5-SATs presented in Table 1 have been reported previously.^47,48^ Affinities of 5-SATs and 5-HT_1_ reference compounds were evaluated by radioligand competition binding assays using [^3^H]5-CT as the radioligand for 5-HT_1A_, 5-HT_1B_, and 5-HT_1D_ receptors and [^3^H]5-HT for the 5-HT_1F_ receptor (Table 1). The *K*_D_ values for [^3^H]5-CT at 5-HT_1A_, 5-HT_1B_, 5-HT_1D_ were 2.5 ± 0.2, 5.3 ± 0.5, and 0.82 ± 0.1 nM, respectively; the *K*_D_ for [^3^H]5-HT at 5-HT_1F_ was 7.8 ± 2.3 nM.

Regardless of the stereochemistry or substitution at the C(2) and C(5) positions, 5-SATs showed at least 100-fold selectivity for 5-HT_1A_, 5-HT_1B_, and 5-HT_1D_ receptors over the 5-HT_1F_ receptor, as did reference 5-HT_1_ receptor agonists 8-OH-DPAT, NLX-112, buspirone, and aripiprazole. LY-334370 (Figure 1) demonstrated high affinity at the 5-HT_1F_ receptor but low affinity at 5-HT_1A_, 5-HT_1B_, and 5-HT_1D_ receptors, consistent with literature reports.^39^

Among 5-SATs presented in Table 1, (*S*)-stereochemistry at the C(2) position conferred a higher affinity (35- to 1000-fold) than the (*R*)-configuration at 5-HT_1A_, 5-HT_1B_, and 5-HT_1D_ receptors regardless of C(2) or (5)-substitution. All 5-SATs have a C(2) substituent that contains a basic nitrogen, which is typical among aminergic neurotransmitter GPCR ligands and generally essential for interaction with the highly conserved aspartic acid residue at position 3.32 (i.e., D3.32).^50^ 5-SAT stereoselective binding at 5-HT_1_ receptors suggests that C(2) stereochemistry is an important molecular determinant for receptor recognition. Interestingly, the 5-HT_1A_ receptor reference agonist 8-OH-DPAT showed no stereochemical preference at 5-HT_1A_ receptors, and, in contrast to 5-SAT type aminotetralins, it showed (2*R*)- over (2*S*) stereoselectivity at 5-HT_1B_ and 5-HT_1D_ receptors, though, with 10–20 fold lower affinity than either enantiomer at the 5-HT_1A_ receptor.

5-SAT analogues with a C(2) *N*,*N*-dimethylamine substituent demonstrated highest affinity at the 5-HT_1D_ receptor regardless of the C(5) substituent. However, C(5) substitution impacted secondary binding preference for either 5-HT_1A_ or 5-HT_1B_ receptors. For example, when C(5) was phenyl (**5-PAT**) or 2′-fluorophenyl (**FPT**), there was no difference between affinities at 5-HT_1A_ and 5-HT_1B_ receptors; however, for the 2′-chlorophenyl analogue (**CPT**), there was a preference for 5-HT_1B_ over 5-HT_1A_ receptors. Substituting the C(5) position with various 5-membered heteroaromatic systems (**FAT**, **TAT**, **NMP**) did not produce significant differences in affinity between analogues at any of the 5-HT_1_ subtypes, that is, there was no high selectivity for any 5-HT_1_ subtype. Increasing the size of the C(5) aromatic substituent to naphthyl (**NAP**), however, resulted in high affinity at the 5-HT_1B_ and 5-HT_1D_ receptors and low affinity at the 5-HT_1A_ receptor. These results have been probed further in molecular docking and mutagenesis studies, below.

Substituting the 5-SAT scaffold with an *N*,*N*-dipropylamine moiety at the C(2) position produced higher affinity across 5-HT_1_ receptor subtypes compared to analogues with a corresponding dimethylamine moiety. Furthermore, for the C(2) *N*,*N*-dipropylamine 5-SATs, when C(5) was substituted with a 2′-halophenyl moiety, a large 2′-chloro substituent (**DCPT**) provided selectivity for the 5-HT_1B_ receptor over the 5-HT_1A_ receptor. In contrast, a smaller 2′-fluoro substituent (**DFPT**) gave about equipotent results at 5-HT_1A_ and 5-HT_1B_ receptors, analogous to dimethylamine analogues **FPT** and **CPT**. Notably, all the 2′-halophenyl analogues had high affinity at 5-HT_1D_ receptors.

When the 5-SAT C(2) position was substituted with the sterically constrained and relatively large pyrrolidine moiety (**PFPT**), there was about 10-fold higher affinity at the 5-HT_1A_ receptor over 5-HT_1B_ and 5-HT_1D_ receptors. Similarly, the reference agonist NLX-112, with a sterically large and constrained piperidine-type amine moiety, had very high affinity and selectivity at the 5-HT_1A_ receptor over 5-HT_1B_ and 5-HT_1D_ receptors. The piperazine moieties present in buspirone and aripiprazole also likely contributed to their high affinity and selectivity at the 5-HT_1A_ receptor. The impact of ligand steric parameters on accessibility to the critical conserved D3.32 residue in 5-HT_1A_ vs 5-HT_1B_ and 5-HT_1D_ receptors has been studied further in molecular modeling and mutagenesis studies, below. Representative dose–response binding curves of lead compounds are shown in the Supporting Information (Figure S1).

### Effects of 5-SATs and Reference Ligands on cAMP Accumulation in HEK_t_ Cells Expressing 5-HT_1_ Receptor Subtypes

The (2*S*)-enantiomers of 5-SATs presented in Table 1 all had reasonably high 5-HT_1_ receptor affinity and thus were assessed for canonical function at 5-HT_1A_, 5-HT_1B_, and 5-HT_1D_ receptors individually expressed in HEK293T cells, via measurement of cAMP accumulation using a time-resolved fluorescence resonance energy transfer (TR-FRET) immunoassay. The nonselective 5-HT_1A_, 5-HT_1B_, and 5-HT_1D_ receptor full agonist 5-CT, (tritiated version used as radioligand), a stable analogue of 5-HT, was used as a positive control and to define maximum efficacy (*E*_MAX_). In functional assays, 5-CT showed potency and efficacy similar to 5-HT at 5-HT_1A_, 5-HT_1B_, and 5-HT_1D_ receptors (Figure S2).

All (2*S*)-5-SATs tested were agonists at 5-HT_1A_, 5-HT_1B_, and 5-HT_1D_ receptors (Table 2) and C(2) and/or C(5) substitution impacted agonist potency and efficacy at 5-HT_1_-subtypes. For example, among 5-SATs substituted with *N*,*N*-dimethylamine at the C(2) position, the 5-phenyl substituted analogue **5-PAT** was over 10-fold more potent at the 5-HT_1D_ receptor compared to 5-HT_1A_ and 5-HT_1B_ receptors and was a full agonist at 5-HT_1A_ and 5-HT_1D_ receptors (*E*_MAX_ > 90%) but a weak partial agonist (*E*_MAX_ ∼ 30%) at the 5-HT_1B_ receptor. The 5-(2′-halophenyl) analogues **FPT** and **CPT**, however, were high-potency, full-efficacy (*E*_MAX_ > 90%) agonists at the 5-HT_1B_ receptor. When the 5-SAT C(2) group was *N*,*N*-dipropylamine, there were no potency and efficacy differences between 5-phenyl and 5-(2′-halophenyl) analogues (**DPAT**, **DFPT**, **DCPT**) at 5-HT_1A_ and 5-HT_1D_ receptors. At the 5-HT_1B_ receptor, however, **DPAT** displayed low potency and weak partial agonist activity (*E*_MAX_ ∼ 20%), while **DFPT** and **DCPT** were high-potency, full-efficacy (*E*_MAX_ > 90%) agonists at the same receptor. These results suggest that the C(2) and C(5) substituents on the 5-SAT scaffold impact binding pocket interactions differentially, depending on the 5-HT_1_ subtype, as was borne out in molecular modeling and mutagenesis studies, below.

**Figure 2:**
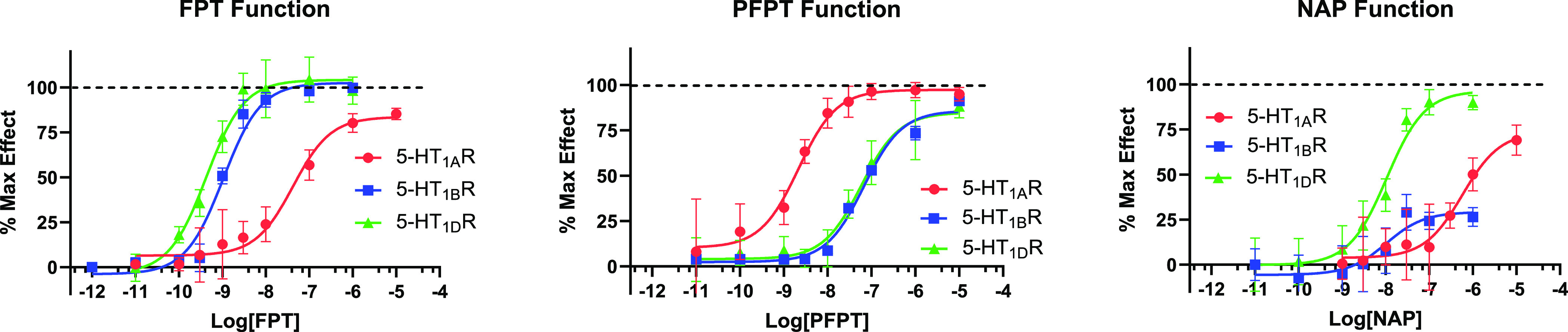
Dose–response curves of (S)FPT (A), (S)PFPT (B), and (S)NAP and (C) in vitro function at 5-HT_1A_, 5-HT_1B_, and 5-HT_1D_ receptors.

When the (2*S*)-5-SAT C(2) position was substituted with pyrrolidine and C(5) was substituted with 2′-fluorophenyl (**PFPT**), there was selective (∼40-fold) high potency at the 5-HT_1A_ receptor (Figure 2). In contrast, when C(2) was *N*,*N*,-dimethyl amine and C(5) was 2′-fluorophenyl (**FPT**), there was selective (∼40-fold) high potency at 5-HT_1B_ and 5-HT_1D_ receptors (Figure 2). These ligand structure–function results have been explored in molecular docking and mutagenesis studies, below.

(2*S*)-5-SATs with *N*,*N*,-dimethylamine at C(2) and substituted with different aromatic groups at the C(5)-position had different potencies and efficacies at 5-HT_1_ subtypes. For example, when C(5) was naphthyl (**NAP**), there was higher potency at 5-HT_1B_ and 5-HT_1D_ receptors compared to the 5-HT_1A_ receptor (Figure 2), consistent with affinity results. However, like other 5-SATs without a 2′-halogen-containing C(5) moiety, **NAP** was a weak partial agonist at 5-HT_1B_ receptors but full efficacy agonist at 5-HT_1A_ and 5-HT_1D_ receptors. When the C(5) aromatic group was furanyl (**FAT**), high potency, efficacy, and selectivity was observed at the 5-HT_1D_ receptor. In contrast, when the C(5) aromatic group was thienyl (**TAT**), there was selective high potency and efficacy at 5-HT_1A_ and 5-HT_1B_ receptors compared to the 5-HT_1D_ receptor. Meanwhile, the C(5) *N*-methylpyrrole analogue (**NMP**) was the only (2*S*)-5-SAT compound with partial agonist activity (*E*_max_ < 85%) across all 5-HT_1_ subtypes. While it was possible to rationalize the impact of the size of the C(5) aromatic substituent (see molecular modeling and mutagenesis results for **NAP**, below), there was no clarity about the impact of the type of aromatic substituent, that is, naphthyl, furanyl, thienyl, and *N*-methylpyrrole.

Functional potencies of reference compounds were consistent with affinity potencies. For example, (*R*)- and (*S*)-8-OH-DPAT had equipotent functional potency at 5-HT_1A_ receptors, but the (*R*)-enantiomer was more potent at 5-HT_1B_ and 5-HT_1D_ receptors. Buspirone displayed partial agonism at 5-HT_1A_, 5-HT_1B_, and 5-HT_1D_ receptors and was far more potent at the 5-HT_1A_ receptor. NLX-112 was a highly selective full agonist at 5-HT_1A_ receptors, with no discernible functional response at 10 μM at 5-HT_1B_ and 5-HT_1D_ receptors. Similarly, aripiprazole was a highly potent and efficacious 5-HT_1A_ receptor agonist but had low potency at 5-HT_1B_ and 5-HT_1D_ receptors.

### Interactions of **FPT**, **PFPT**, **NAP**, and 5-CT at 5-HT_1A,_ 5-HT_1B_ and 5-HT_1D_ Receptor Models after MD Simulations

The results above suggest that 5-SAT affinity and functional potency and efficacy at 5-HT_1_ receptor subtypes are impacted by type of substituents at the C(2) and C(5) positions. Although 5-HT_1_ subtypes share a high degree of TM (transmembrane) sequence homology,^51^ there are several differences in binding pocket amino acid composition (Table S1) and side chain orientation, which could account for differential 5-SAT activity at 5-HT_1_ subtypes. To rationalize binding and function results as well as guide development of ligands optimized to exploit SAR results above, molecular docking and MD were undertaken for **FPT**, **PFTP**, and **NAP** at 5-HT_1A,_ 5-HT_1B_ and 5-HT_1D_ receptor models. Additionally, we docked 5-CT at each receptor subtype to contextualize 5-SAT affinity results, which were determined via displacement of [^3^H]5-CT (Tables S2–S5). Molecular models were built using the reported cryo-EM structures of 5-HT_1A_ (PDB code 7E2Y) and 5-HT_1D_ (PDB code 7E32),^41^ and the crystal structure of 5-HT_1B_ (PDB code 4IAR).^43^ Docking poses were selected based on the lowest XP scores from induced-fit-docking (IFD) using Schrödinger, and followed by MD simulations. MD simulations were run for up to 1 μs to enhance the likelihood of capturing the full receptor conformational change stabilized by agonist ligand binding, which is believed to require several hundred nanoseconds.^52^

**Figure 3:**
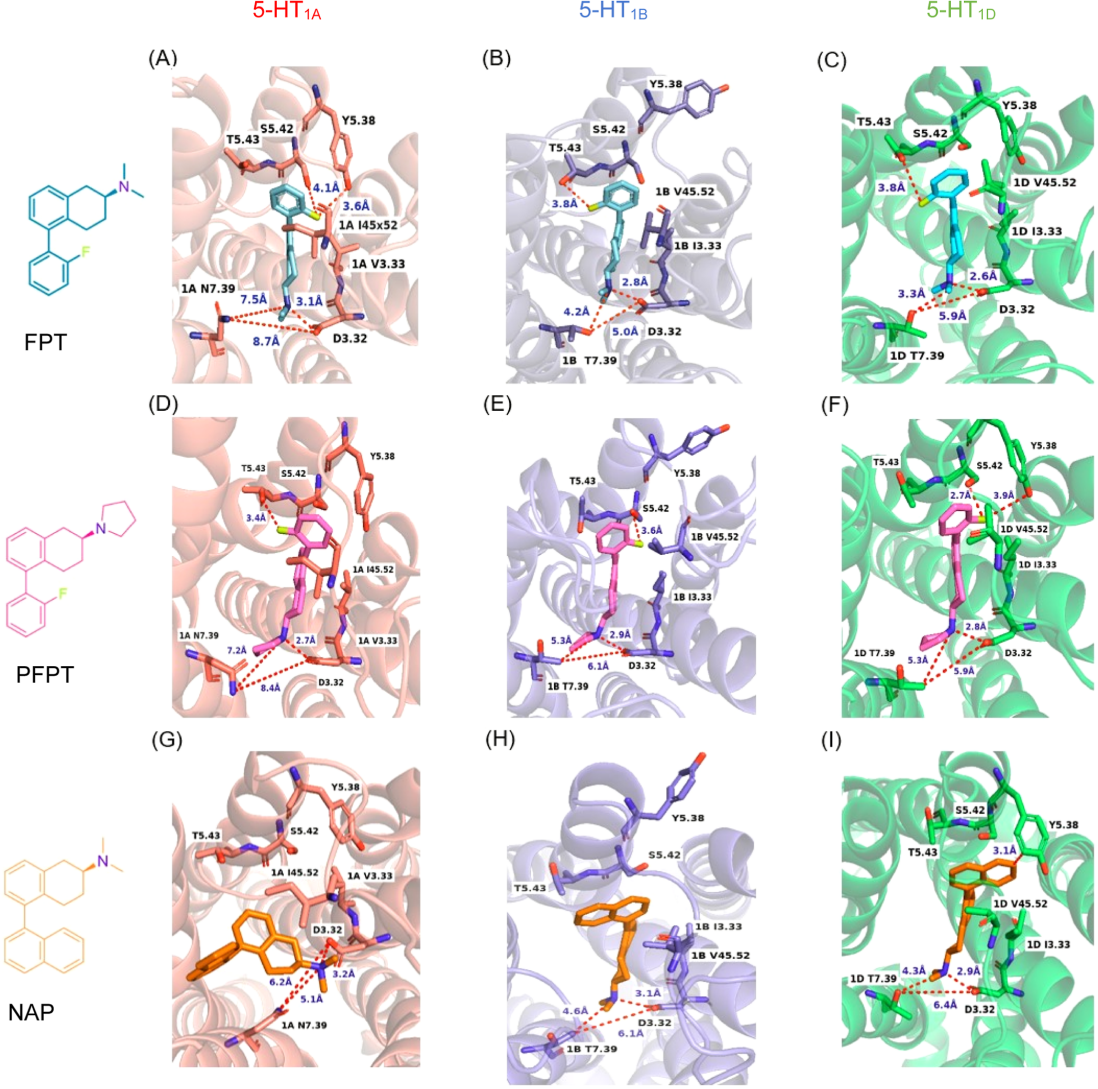
Ligand molecular docking studies (the 5-HT_1A_ model binding pocket is displayed in salmon color, 5-HT_1B_ is displayed in blue, and 5-HT_1D_ is displayed in green). Shown are final docking poses of **FPT** (teal) at (A) 5-HT_1A_, (B) 5-HT_1B_, and (C) 5-HT_1D_; **PFPT** (pink) at (D) 5-HT_1A_, (E) 5-HT_1B_, and (F) 5-HT_1D_; **NAP** (orange) at (G) 5-HT_1A_, (H) 5-HT_1B_, and (I) 5-HT_1D_.

Following MD simulations, the **FPT** C(2) *N*,*N*-dimethylamine moiety interacted closely with conserved residue D3.32 at distances of 3.1, 2.8, and 2.6 Å at the 5-HT_1A_, 5-HT1_1B_, and 5-HT_1D_ receptor models, respectively (Figure 3A–C), apparently forming an ionic interaction that is critical for aminergic GPCR ligands.^50^ The C(2) pyrrolidine moiety of **PFPT** (Figure 3D–F) and the C(2) *N*,*N*-dimethylamine moiety of **NAP** (Figure 2G–I) also docked close enough (<3.5 Å) to D3.32 to form an ionic bond at the 5-HT_1A_, 5-HT_1B_, and 5-HT_1D_ receptor models.

At the 5-HT_1A_ receptor, the C(2) amine moieties of **FPT** (Figure 3A) and **PFPT** (Figure 3D) orient between TMs 3 and 7 with similar distances from residue N7.39 (7.5 Å for **FPT** and 7.2 Å for **PFPT**). Notably, there is a relatively large space between 5-HT_1A_ residues D3.32 and N7.39, that is, 8.7 Å for the **FPT** dock and 8.4 Å for the **PFPT** dock. In contrast, at the 5-HT_1B_ and 5-HT_1D_ receptor models, **FPT** and **PFPT** dock such that there is a smaller space between D3.32 and T7.39, that is, 5.0 Å for **FPT** and 6.1 Å for the **PFPT** at 5-HT_1B_, and 5.9 Å for both **FPT** and **PFPT** at 5-HT_1D_. It appears that when the large space between 5-HT_1A_ TMs 3 and 7 is engaged with a large basic C(2) amine group, such as the pyrrolidine moiety of **PFPT** versus the smaller dimethylamine moiety of **FPT**, affinity and functional potency is increased, providing selectivity for the 5-HT_1A_, that is, for **PFPT**, there was 10-fold higher affinity and 35-fold higher potency at 5-HT_1A_ over 5-HT_1B_ and 5-HT_1D_ receptors, whereas for **FPT**, there was higher affinity (2- and 4-fold, respectively) and functional potency (about 30- and 80-fold, respectively) at 5-HT_1B_ and 5-HT_1D_ over 5-HT_1A_ receptors (Tables 1 and 2). **NAP**, which like FPT has a relatively small C(2) dimethylamine moiety, also had higher affinity (10- and 70-fold, respectively) and functional potency (about 10- and 40-fold, respectively) at 5-HT_1B_ and 5-HT_1D_ over 5-HT_1A_ receptors.

At the 5-HT_1A_ receptor, the **FPT** C(5) 2′-fluorophenyl moiety docked near residues Y5.38 (3.6 Å) and S5.42 (4.1 Å) (Figure 3A), apparently via halogen binding. At the 5-HT_1B_ and 5-HT_1D_ receptors, the **FPT** 2′-fluorine moiety oriented ∼180° in the opposite direction and docked close (3.8 Å) to the conserved residue T5.43 (Figure 3B,C), which perhaps accounted for the higher affinity and functional potency observed for **FPT** at the 5-HT_1B_ and 5-HT_1D_ receptors.

Interactions involving TM5 essentially were reversed for **PFPT** compared to **FPT**, that is, at the 5-HT_1A_ receptor, the **PFPT** C(5) 2′-fluorophenyl docked close to T5.43 (3.4 Å) and at the 5-HT_1B_ and 5-HT_1D_ receptor, it docked close to the S5.42 (3.6 Å and 2.7 Å, respectively) as well as close (3.9 Å) to the 5-HT_1D_ Y5.38 hydroxyl group (Figure 3D–F). Notably, the 5-HT_1B_ Y5.38 residue is oriented away from the binding pocket in all docks. The different interactions of **FPT** and **PFPT** with key TM5 residues at the 5-HT_1_ subtypes are consistent with the differential receptor selectivity of **FPT** and **PFPT**, that is, **FPT** is selective for 5-HT_1B_ and 5-HT_1D_ receptors over 5-HT_1A_ receptors and vice versa for **PFPT** (Tables 1 and 2).

At the 5-HT_1A_ receptor, the **NAP** naphthyl moiety oriented differently than at the 5-HT_1B_ and 5-HT_1D_ receptors (Figure 3G–I), likely, due to steric effects of the larger isoleucine residue at ECL position 45.52 in 5-HT_1A_ (valine in 5-HT_1B_ and 5-HT_1D_). At the 5-HT_1A_ receptor, we could not identify close interactions between the C(5) naphthyl moiety and TM 5 amino acids, which perhaps is consistent with the relatively low affinity and functional potency of **NAP** at the 5-HT_1A_ receptor compared to 5-HT_1B_ and 5-HT_1D_ receptors (Tables 1 and 2). To further test the importance of position 45.52 regarding steric interactions with the ligands at 5-HT_1_ receptors, we point-mutated this residue in studies below.

At the 5-HT_1B_ and 5-HT_1D_ receptors (Figure 3H–I), there appear to be π-hydroxyl (hydrogen) electrostatic interactions between the **NAP** 5-naphthyl moiety and S5.42 (2.7 Å) and T5.43 (3 Å) at the 5-HT_1B_ receptor (Figure 3G), whereas there likely are π–π stacking interactions with the phenyl moiety of Y5.38 (3.1 Å) at the 5-HT_1D_ receptor (Figure 3I); these differential interactions involving the **NAP** naphthyl moiety may account for the higher affinity and functional potency of **NAP** observed at 5-HT_1B_ and 5-HT_1D_ receptors compared to the 5-HT_1A_ receptor (Tables 1 and 2). Docking results for **NAP** were validated by point mutation of the residue at the Y5.38 position in mutagenesis studies, below.

The nonselective full agonist 5-CT showed little differences among final docked poses at 5-HT_1A_, 5-HT_1B_, and 5-HT_1D_ receptors (Figure S3). At all three 5-HT_1_ receptor models, the primary amine moiety docked close (<3.5 Å) to conserved residue D3.32, apparently forming an ionic bond. The 5-CT carboxamide group appeared to interact with the three conserved amino acids Y5.38, S5.42, and T5.43 at the 5-HT_1A_, 5-HT_1B_, and 5-HT_1D_ receptor models. Importantly, these four conserved amino acids (D3.32, Y5.38, S5.42, and T5.43) also played a role in molecular docking results for the 5-SATs (Figure 3).

### Mutagenesis Studies

**Figure 4:**
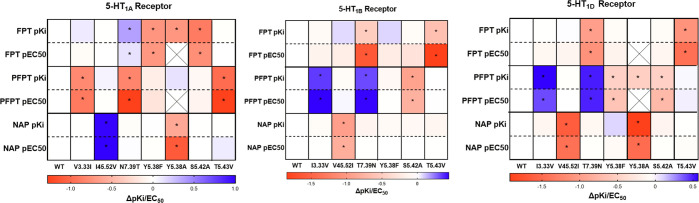
Influence of 5-HT_1A_, 5-HT_1B_, and 5-HT_1D_ receptor point mutations on the affinity (p*K*_i_) and functional potency (pEC_50_) of **FPT**, **PFPT**, and **NAP**. Heat maps indicate change (vs WT receptors) in affinity (p*K*_i_) and functional potency (pEC_50_) of **FPT**, **PFPT**, and **NAP** at point-mutated 5-HT_1_-type receptors. (*) denotes significant difference from WT as determined by Student’s *t*-test, *P* < 0.05. An “X” through a box indicates that the experiment was not done.

To validate molecular docking results and provide insights into 5-SAT molecular determinants for 5-HT_1_ subtype-selective affinity and function, we conducted experimental binding and functional studies of **FPT**, **PFPT**, **NAP**, and 5-CT at point-mutated 5-HT_1A_, 5-HT_1B_, and 5-HT_1D_ receptors. To ensure that functional and binding results could be compared across mutated 5-HT_1A_, 5-HT_1B_, and 5-HT_1D_ receptors, we analyzed basal and max effect (i.e., response of 10 μM 5-CT) cAMP levels at each receptor construct (Figure S4). We found that there was no statistical difference between wild-type (WT) and mutant basal values as well as WT and mutant max effect. Generally, at point-mutated receptors wherein there was a significant change in affinity for a ligand, there also was a corresponding significant change in functional potency (Figure 4). The p*K*_i_ and pEC_50_ values for **FPT**, **PFPT**, and **NAP** at each point mutated receptor are in Table S2.

To test the hypothesis that selectivity for binding at the 5-HT_1A_ receptor can be realized by exploiting the sterically generous space around residue D3.32 at the 5-HT_1A_ receptor compared to 5-HT_1B_ and 5-HT_1D_ receptors, we assessed affinity and function of **FPT** at the N7.39T 5-HT_1A_ receptor wherein it was hypothesized that the binding pocket area around D3.32 would be more similar to the 5-HT_1B_ and 5-HT_1D_ receptors. Results showed that **FPT** had a significantly higher affinity and functional potencies at the N7.39T 5-HT_1A_ receptor compared to the WT receptor and comparable to its affinity and functional potencies at WT 5-HT_1B_ and 5-HT_1D_ receptors. Analogously, **FPT** had a significantly lower affinity and functional potency at T7.39N 5-HT_1B_ and 5-HT_1D_ receptors compared to the corresponding WT receptors, similar to its potencies at the WT 5-HT_1A_ receptor. Meanwhile, **PFPT**, which had high selectivity for binding and function at WT 5-HT_1A_ over WT 5-HT_1B_ and 5-HT_1D_ receptors, had significantly reduced affinity and functional potency at the T7.39N 5-HT_1A_ receptor, comparable to its potencies at the WT 5-HT_1B_ and 5-HT_1D_ receptors. At N7.39T 5-HT_1B_ and 5-HT_1D_ receptors, **PFPT** had a significantly higher affinity and functional potencies compared to WT receptors, similar to the values of **PFPT** at the WT 5-HT_1A_ receptor. Thus, nonconserved amino acid residues at position 7.39 were a key determinant for 5-SAT selectivity at 5-HT_1A_ subtypes.

To further probe the steric interactions around D3.32, we tested the affinity and function of **FPT** at the V3.33I 5-HT_1A_, I3.33V 5-HT_1B,_ and I3.33V 5-HT_1D_ receptors. Interestingly, **FPT** potencies at the V3.33I 5-HT_1A_ receptor were not significantly different compared to values at WT 5-HT_1A_ receptors. Furthermore, there were no significant changes in **FPT** potencies at the I3.33V compared to WT 5-HT_1B_ and 5-HT_1D_ receptors. Thus, position 3.33 does not seem to impact **FPT** selectivity at 5-HT_1_ receptor subtypes.

In contrast, for **PFPT**, both the affinity and functional potencies were significantly reduced at the V3.33I 5-HT_1A_ receptor compared to the WT receptor and comparable to its potencies at WT 5-HT_1B_ and 5-HT_1D_ receptors. Analogously, affinity and functional potencies of **PFPT** were significantly increased at I3.33V 5-HT_1B_ and 5-HT_1D_ receptors, displaying values comparable to WT 5-HT_1A_ receptors. These results indicate that position 3.33 impacts **PFPT** selectivity at 5-HT_1_ receptor subtypes. Thus, it appears that larger C(2) substituents on the 5-SAT scaffold, such as the pyrrolidine moiety of **PFTP** compared to the dimethylamine group of **FPT**, can impact affinity and functional selectivity at 5-HT_1_ receptor subtypes via steric interactions with the residue at position 3.33.

Notably, molecular modeling results indicated that at the 5-HT_1A_ receptor, the **FPT** fluorine atom interacts with the hydroxyl groups on Y5.38 and S5.42 (Figure 3A). To validate the molecular dock, we assessed **FPT** affinity and function at the Y5.38F and at the S5.42A 5-HT_1A_ receptors and found there were significant decreases in affinity and potency compared to the WT receptor. Analogously, modeling results indicated that at the 5-HT_1B_ and 5-HT_1D_ receptors, the **PFPT** fluorine atom interacts with the hydroxyl groups on Y5.38 (only at 5-HT_1D_) and S5.42 (Figure 3E,F). To validate the molecular dock, we assessed **PFPT** affinity and function at the Y5.38F 5-HT_1B_ and at the S5.42A 5-HT_1D_ receptors and found there were significant decreases in affinity and potency compared to the corresponding WT receptors.

We also tested the hypothesis that at 5-HT_1B_ and 5-HT_1D_ receptors, the **FPT** fluorine atom interacts with the T5.43 hydroxyl group (Figure 3D–F) by assessing the **FPT** affinity and function at the T5.43V 5-HT_1B_ and the T5.43V 5-HT_1D_ receptors. At both mutated receptors, there were significant reductions in **FPT** affinity and functional potencies. Analogously, we tested the hypothesis that at the 5-HT_1A_ receptor, the **PFPT** fluorine atom interacts with the T5.43 hydroxyl group (Figure 3D) by assessing the **PFPT** affinity and function at the T5.43V 5-HT_1A_ receptor. At the T5.43V 5-HT_1A_ receptor, there was a significant reduction in the **PFPT** affinity and functional potencies compared to the WT 5-HT_1_A receptor. Thus, it appears that 5-SATs, which can realize binding interactions with conserved residue T5.43, likely will have higher affinity and functional potencies.

To validate selective binding and functional interactions proposed for **NAP** involving Y5.38 at the WT 5-HT_1D_ receptor, we assessed **NAP** affinity at the Y5.38A 5-HT_1D_ receptor. The elimination of potential π–π interactions between the **NAP** naphthyl moiety and position 5.38 at the Y5.38A 5-HT_1D_ receptor resulted in a significant decrease of **NAP** affinity and functional potencies compared to the 5-HT_1D_ WT receptor, apparently validating the molecular docking results (Figure 3I). We also assessed **NAP** affinity and function at the Y5.38F 5-HT_1D_ receptor and found there was no difference in **NAP** affinity and functional potencies compared to the WT 5-HT_1D_ receptor, further suggesting that the **NAP** interaction primarily is via π–π interaction with the with Y5.38 phenyl moiety rather than π–hydrogen interaction with the hydroxyl moiety. Finally, we assessed affinity and function of **NAP** at the I45.52V 5-HT_1A_ receptor wherein it was hypothesized that the smaller valine residue (as exists in the 5-HT_1B_ and 5-HT_1D_ receptor binding pockets) could accommodate productive binding interactions with **NAP**. At I45.52V 5-HT_1A_ receptors, **NAP** had increased affinity and functional potency compared to WT 5-HT_1A_ receptors, similar to its affinity at WT 5-HT_1B_ and 5-HT_1D_ receptors. Analogously, at the V45.52I 5-HT_1B_ and 5-HT_1D_ receptors, there was a significant reduction in **NAP** affinity and functional potency compared to the corresponding WT receptors and similar to the WT 5-HT_1A_ receptor, indicating that 5-SAT steric interactions with the residue at 45.52 impacts the 5-HT_1_ receptor subtype selectivity.

We also validated the binding poses of 5-CT at 5-HT_1_ subtypes (Figure S3) by assessing their affinity and function at point-mutated subtypes involving the same receptor positions as above, that is, 3.33, 45.52, 7.39, 5.38, 5.43, and 5.43. There was a significant reduction in 5-CT affinity (Tables S3) and functional potency (Figure S5) only at receptor constructs which contained a conserved amino acid mutation, that is, Y5.38F, Y5.38A, S5.42A, and T5.43V 5-HT_1A_ and 5-HT _1D_ receptors; S5.42A and T5.43V 5-HT_1B_ receptors. These results suggest a common mechanism for 5-CT activation of 5-HT_1A_, 5-HT_1B_, and 5-HT_1D_ receptors that involves these conserved amino acid residues. Unsurprisingly, mutagenesis work from other groups have shown that both S5.42 and T5.43 also are critical for 5-HT binding and function.^53^ Notably, as concluded above, molecular docking and mutagenesis studies indicated that Y5.38, S5.42, and T5.43 also are key molecular determinants for the 5-SAT (**FPT**, **PFPT**, **NAP**) agonist function as well as 5-HT_1_ receptor subtype selectivity. Based on results herein, it appears that sterically large ligands such as the 5-SATs as well as the reference agonists (buspirone, NLX-112, aripiprazole) can realize 5-HT_1_ receptor subtype selectivity not possible with small flexible agonist ligands such as 5-CT and 5-HT. Representative normalized dose–response curves of FPT at pointed-mutated 5-HT_1A_, 5-HT_1B_, and 5-HT_1D_ receptors can be found in the Supporting Information (Figure S6).

### Design and Synthesis of 5-SATs to Exploit 5-HT_1_ Receptor Subtype-Selective Interactions

**Figure 5:**
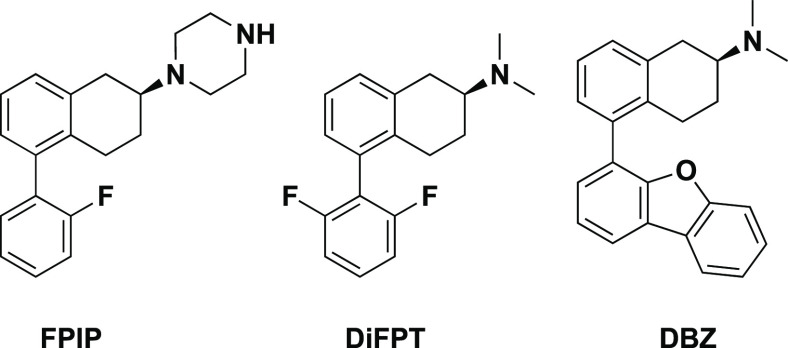
Chemical structures of novel 5-SATs (2*S*)-**FPIP**, **DiFPT**, and **DBZ**.

Based on 5-SAT SAR, molecular docking, and mutagenesis results discussed above, we undertook synthesis of additional (2*S*)-5-SATs (Figure 5) designed to target activation of specific 5-HT_1_ receptor subtypes.

**Scheme 1:**
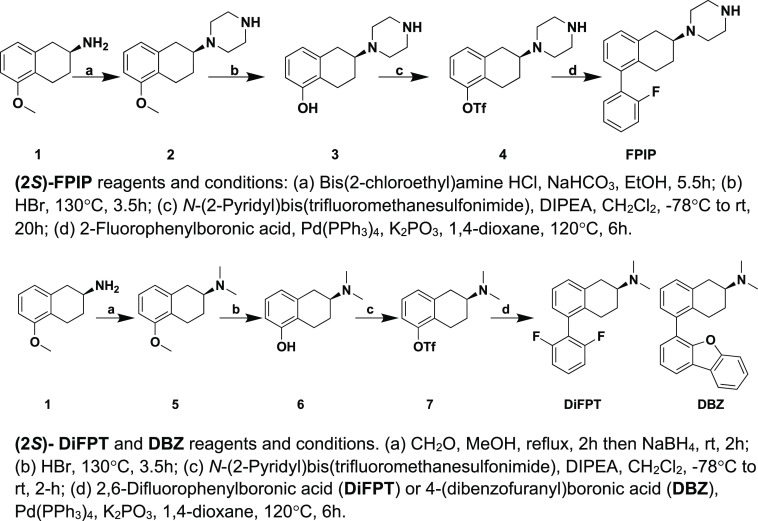
Synthesis of (2*S*)-**FPIP**, **DiFPT**, and **DBZ**

As noted above, (2*S*)-**FPT** had moderately selective affinity and agonist potency at 5-HT_1B_ and 5-HT_1D_ receptors over the 5-HT_1A_ receptor (Tables 1 and 2). It was hypothesized that substituting the **FPT** dimethylamine moiety with the sterically larger piperazine group present in 2*S*-**FPIP** (Figure 4) could selectively occupy the generous space around D3.32 at the 5-HT_1A_ receptor binding pocket, but which is more restricted at 5-HT_1B_ and 5-HT_1D_ receptors (Figure 3). A stereoselective synthetic scheme (Scheme 1) was utilized to synthesize (2*S*)-**FPIP** via cyclization of (2*S*)-5-methoxy-1,2,3,4-tetrahydronaphthalen-2-amine HCl **1** with bis(2-chloroethyl)amine HCl^54^ to give compound **2**, which was demethylated (**3**) and then reacted with *N*-(2-pyridyl)-bis(trifluoromethanesulfonimide) to form triflate **4**. Compound **4** was coupled with 2-fluorophenylboronic acid via a Suzuki–Miyaura reaction to give (2*S*)-**FPIP** (Figure 5 and Scheme 1).

As summarized in Table 3, (2*S*)-**FPIP** had high selectivity for binding and activation of 5-HT_1A_ over 5-HT_1B_ and 5-HT_1D_ receptors. It is noted that the 5-HT_1A_ receptor selective affinity and functional potency of (2*S*)-**FPIP**, apparently associated with its large C(2) piperazine moiety, also is displayed by (2*S*)-**PFPT** (Tables 1 and 2), which has a relatively large basic pyrrolidine moiety at C(2). Likewise, 5-HT_1A_-selective interactions (Tables 1 and 2) were observed for the reference 5-HT_1_ agonists aripiprazole and buspirone, which also have a large basic piperazine moiety, as well as for NLX-112, which has a large pyridinyl moiety attached to its basic alkyl amine group.

**Table 3:** Affinity (p*K*_i_), Functional Potency (pEC_50_), and Efficacy (*E*_MAX_) of Novel 5-SATs at 5-HT_1A_,5-HT_1B_, and 5-HT_1D_ Receptors

	**5-HT**_**1A**_			**5-HT**_**1B**_			**5-HT**_**1D**_		
**(2***S***)-5-SAT**	p*K*_i_	pEC_50_	*E*_MAX_ (%)	p*K*_i_	pEC_50_	*E*_MAX_ (%)	p*K*_i_	pEC_50_	*E*_MAX_ (%)
**FPIP**	9.01 ± 0.09	8.92 ± 0.15	95 ± 2	6.97 ± 0.13	6.90 ± 0.10	99 ± 3	7.06 ± 0.14	6.83 ± 0.05	98 ± 2
**Di-FPT**	8.77 ± 0.19	8.76 ± 0.10	98 ± 2	8.79 ± 0.07	8.68 ± 0.03	100 ± 1	8.85 ± 0.13	8.78 ± 0.24	100 ± 1
**DBZ**	6.51 ± 0.04	6.09 ± 0.02	93 ± 4	7.16 ± 0.05	7.19 ± 0.09	93 ± 2	8.32 ± 0.04	8.36 ± 0.13	99 ± 2

Regarding the C(5) position of 5-SATs, it was noted that the fluorine moiety of (2*S*)-**FPT** interacted with conserved residues Y5.38 and S5.42 at the 5-HT_1A_ receptor but with conserved residue T5.43 at the 5-HT_1B_ and 5-HT_1D_ receptors (Figure 3A–C), where it has higher affinity and functional potency (Tables 1 and 2). Analogously, the fluorine of (2*S*)-**PFPT** interacted closely with T5.43 at the 5-HT_1A_ receptor where it has higher affinity and functional potency compared to 5-HT_1B_ and 5-HT_1D_ receptors, where the 2-fluorophenyl moiety interacts with Y5.38 and S5.42 (Tables 1 and 2; Figure 3D–F). These results suggest that 5-SAT C(5) substituents, which can realize halogen-hydroxyl interaction with T5.43 at 5-HT_1_ receptor subtypes, can translate to high affinity and agonist potency. To test this hypothesis, we synthesized (2*S*)-**Di-FPT** (Figure 5 and Scheme 1) with the rationale to increase the halogen binding potential with 5-HT_1_ conserved residue T5.43. As shown in Scheme 1, trifilate **7** was coupled with 2,6-difluorophenylboronic acid via a Suzuki–Miyaura reaction to give (2*S*)-**Di-FPT**. As shown in Table 3, (2*S*)-**Di-FPT** had nonselective but high affinity and agonist potency across receptor subtypes (Table 3).

As observed in Figure 3G–I, when the C(5) substituent is the large naphthyl aromatic moiety in (2*S*)-**NAP**, there were apparent π–π interactions with Y5.38 at the 5-HT_1D_ receptor, which resulted in significantly higher affinity and agonist potency compared to the 5-HT_1A_ receptor, where we did not observe productive interactions concerning the NAP naphthyl moiety. In contrast, at the 5-HT_1B_ receptor, the (2*S*)-**NAP** naphthyl moiety appeared to interact with S5.42 and T5.43 and the potency was less than at the 5-HT_1D_ receptor. To potentially maximize C(5) aromatic interactions with S5.42 and T5.43 (5-HT_1B/1D_) and Y5.38 (5-HT_1D_), we substituted the naphthyl moiety of (2*S*)-**NAP** with the larger dibenzofuranyl moiety present in (2*S*)-**DBZ** (Figure 5 and Scheme 1), with the expectation that productive binding interactions would be realized at the 5-HT_1D_ receptor. As shown in Scheme 1, **7** was coupled with 4-(dibenzofuranyl)boronic acid via a Suzuki–Miyaura reaction to give (2*S*)-**DBZ**. As shown in Table 3, the (2*S*)-**DBZ** analogue had higher affinity and functional potency at 5-HT_1B_ and 5-HT_1D_ receptors compared to 5-HT_1A_ receptors and was the most 5-HT_1D_-selective 5-SAT synthesized in this work. Future studies will explore the 5-HT_1_ pharmacology of 5-SATs with C(5) aromatic moieties even larger than dibenzofuran such as anthracene.

## Conclusions

In this work, we delineated molecular determinants for 5-HT_1_ receptor subtype pharmacology of 5-SAT analogues substituted at the C(2) and C(5) positions. We conclude that 5-SAT interactions at 5-HT_1_ subtype positions 5.38, 5.42, and 5.43 likely are involved in receptor activation, and, together with residues at positions 3.33 and 7.39, likely are involved in binding and functional potencies to impact subtype selectivity. We designed and synthesized new 5-SATs to exploit the results obtained from experimental and computational studies herein and realized a new full efficacy 5-HT_1A_ agonist (**FPIP**) with 100-fold selectivity over 5-HT_1B_ and 5-HT_1D_ receptors. We will continue efforts to develop highly selective ligands for 5-HT_1B_ as well as 5-HT_1D_ receptors using the 5-SAT platform given the chemotype, as represented by (2*S*)-**FPT**, appears to be safe as well as effective, for example, in animal models of substance use disorder^55^ as well as autism and fragile X syndrome.^44−46^

## Methods

### Cell Culture and Transfection

Cell Culture techniques followed procedures previously published by our lab.^44,45,48,56,57^ Briefly, HEK293T cells were obtained from ATTC (Manassas, VA) and cultured in DMEM supplemented with 10% regular FBS (%v/v) and 1% penicillin (%v/v) at 37 °C in a humidified incubator kept at 5% CO_2_. Cells were passaged at 80% confluency up until passage 20. For transfection, media was discarded and cells were washed with 1× PBS and then incubated with a prewarmed cocktail containing 3 mL of Opti-MEM, 3 mL of DMEM supplemented with 10% dFBS (%v/v), and 1% penicillin (%v/v), 10 μg of desired cDNA construct, and 40 μg of PEI MAX per plate of cells.

### cDNA Constructs

pcDNA3.1+ plasmids encoding the WT human 5-HT_1A_ (HTR01A0000), 5-HT_1B_ (HTR01B0000), 5-HT_1D_ (HTR01D0000), and 5-HT_1F_ (HTR01F0000) receptors were purchased from the cDNA Resource Center (Bloomsburg, PA). Site-directed mutant 5-HT_1A_, 5-HT_1B_, 5-HT_1D_ experiments were preformed using 5′-phosphorylated, PAGE purified custom primers in 100 μL of nuclease free water (Life Technologies, Carlsbad CA) and Quikchange Site-Directed Mutagenesis II kit (Agilent, Santa Clara CA) according to the manufacturer’s protocol. PCR conditions for each point mutation are described in more detail in Tables S4–S6. Purified DNA was sequence-validated by Psomagen Inc. (Cambridge, MA).

### Radioligand Binding Assays

48 hours after transfection with the desired receptor construct, cells were isolated for saturation and competition binding assays as previously described.^47,48,57^ Saturation and competition radioligand binding assays were conducted in an assay buffer consisting of 50 mM Tris HCl, 10 mM MgCl_2_, and 0.1 mM EDTA in filtered deionized water. Briefly, saturation binding assays were conducted to determine the *K*_D_ of [^3^H]5-CT at the 5-HT_1A_, 5-HT_1B_, and 5-HT_1D_ receptor constructs, and [^3^H]5-HT was the radioligand for the 5-HT_1F_ receptor. Briefly, 5–10 μg of the receptor protein, as determined by a Thermo Fisher Pierce BCA protein assay kit, was incubated with 8 concentrations of radioligand in triplicate to determine total binding. Nonspecific binding of [^3^H]5-CT was determined with the addition of 10 μM 5-HT and nonspecific binding of [^3^H]5-HT was determined with the addition of 10 μM lasmiditan. For competitive binding assays to determine *K*_i_ of test ligands, assays were performed in quadruplicate with the *K*_D_ concentration of the radioligand and 10 concentrations of unlabeled ligand in a final volume of 250 μL in a 96-well plate. Nonspecific binding was determined as above for saturation assays. Assay conditions for each receptor construct are given in Table S7.

### Function Assays

Potency (pEC_50_) and efficacy (*E*_MAX_) values for compounds at 5-HT_1A_, 5-HT_1B_, and 5-HT_1D_ receptors were assessed using LANCE Ultra cAMP kit (PerkinElmer) following the manufacturer’s guidelines. Briefly, cells transfected with the desired receptor construct and compounds were incubated in the dark for 90 min at 37 °C. After incubation, cAMP detection reagents were added, and TR-FRET was used to quantify the cAMP produced. Assay conditions for each receptor construct are given in Table S8.

### Molecular Modeling

Docks of 5-CT and 5-SAT analogues at 5-HT_1_ receptors were developed following a procedure previously used by our lab.^48,57^ Three-dimensional ligands were constructed in Masetro (Schrodinger, NY) and optimized via an ab initio quantum chemistry method at the HF/6-31G* level, followed by single-point energy calculations of the molecular electrostatic potential for charge fitting with Gaussian 16.^58^ These docks were formulated using the solved cyro-EM structures of 5-HT_1A_ (PDB code 7E2Y) and 5-HT_1D_ (PDB code 7E32) and the solved crystal structure of 5-HT_1B_ (PDB code 4IAR) all in the active state, with omissions of sidechains and loops added and recapitulated using BIOVIA’s Discovery Studio 2017 (Dassault Systems, Waltham, MA). The 5-HT_1B_ crystal structure was chosen over available 5-HT_1B_ bound to the G_ao_ cyro EM structure^41^ due to the noted differences between the structures of 5-HT_1B_ bound to G_ai_. The ligands were docked into the binding sites in the receptors using IFD simulations^59^ (Schrödinger, Inc.). Default parameters were used for IFD simulations. The residues within 5 Å of ligand poses were selected for side chain optimization by prime refinement. The XP scores were used for ranking of the ligand poses, and top 20 poses of the docked ligand were saved for visual inspection and selection. The pose of docked ligands with the lowest docking XP score were selected as predicted poses.

### Molecular Dynamics

Protonation states of the titratable residues in 5-HT_1A/1B/1D_ receptors were calculated at pH = 7.4 via the use of the H++ server (http://biophysics.cs.vt.edu/).^60^ The ligand–receptor complexes identified in the molecular docks were inserted into a simulated lipid bilayer composed of POPC/POPE/cholesterol (2:2:1)^61^ and a water box using the CHARMM-GUI Membrane Builder webserver (http://www.charmm-gui.org).^62^ Sodium chloride (150 mM) as well as neutralizing counterions were applied to the systems. The PMEMD.CUDA program of AMBER 20 was used to conduct MD simulations. The Amber ff14SB, lipid17, and TIP3P force field was used for the receptors, lipids, and water. The parameters of 5-CT, *S*-FPT, *S*-PFPT, and *S*-NAP were generated using general AMBER force field by the Antechamber module of AmberTools 17. The partial charge was determined via the restrained electrostatic potential charge-fitting scheme by ab initio quantum chemistry at the HF/6-31G* level.^58^ Coordinate files and system topology were established using the tleap module of Amber. The systems were energetically minimized by 500 steps (with a position restraint of 500 kcal mol^–1^ Å^–2^) followed by 2000 steps (without position restraint) using the steepest descent algorithm. Heat was then applied to the systems to drive the temperature from 0 to 303 K using Langevin dynamics with a collision frequency of 1 ps-1. Receptor complexes were position-restrained using an initial constant force of 500 kcal mol^–1^ Å^–2^ during the heating process, subsequently diminished to 10 kcal mol^–1^ Å^–2^, allowing the lipid and water molecules free movement. Before the MD simulations, the systems underwent a 5 ns equilibration. Then, MD simulations were conducted for at least 100–1000 ns using hydrogen mass repartitioning and a time step of 4 fs. The simulations were conducted in an isothermal and isobaric nature, with the pressure maintained using an isotropic position scaling algorithm with the pressure relaxation time fixed at 2 ps. Long-range electrostatics were calculated by a particle mesh Ewald method with a 10 Å cutoff.^63^ The coordinates were saved every 100 ps for analysis. All molecular modeling images were created with PyMOL Version 2.0 Schrodinger.

### Data Analysis

All assays were analyzed using model packages present in GraphPad Prism version 9.4.0.

For radioligand binding data, nonspecific binding was subtracted from the total binding to get specific binding. Saturation binding data was fit to the “binding saturation-specific binding with hill slope” model. Competition binding data were fit to the “binding competition-one-site fit *K*_i_” non-linear regression model and transformed to p*K*_i_ values, reported as mean ± SD.

The functional activity of compounds was determined by stimulating cells expressing the desired receptor with FSK and test compound in parallel with 12 known concentrations of cAMP. The known concentrations of cAMP were used to create a standard curve for each experiment that was used to interpolate the unknown values of cAMP in wells with test compounds. To control for variation in cAMP production between assays, results were normalized to basal (0%) and 10 μM of 5-CT (100%). Experiments with point-mutated receptors were done in parallel with the corresponding WT receptor and results were normalized to basal (0%) and 10 μM of 5-CT (100%). The EC_50_ and *E*_MAX_ values were determined using the “dose response – log[agonist] vs response (three parameter)” nonlinear regression model in Prism and transformed to pEC_50_ values, reported as mean ± SD.

### Statistical Analysis

Unpaired parametric *t*-tests were used to compare results and determine the statistical significance. Statistical analysis was conducted using GraphPad Prism Ver 9.4.0. For our studies *p* < 0.05 was considered statically significant. Details of statistics for experiments can be found in the figure legends.

### Synthesis of 5-SATs

Synthesis of 5-SAT analogues in Table 1 have been previously reported.^47,48^ All reactions were performed under an inert atmosphere of anhydrous nitrogen. Final compounds were converted to their corresponding HCl salts utilizing the 2 M HCl ester, as noted below. All NMR spectra were recorded by Bruker ADVANCE 500 MHz NMR in CDCl_3_ and are expressed as chemical shift (δ) values in parts per million (ppm). Coupling constants (*J*) are presented in Hertz. Abbreviations used in the reporting of NMR spectra include s = single, d = doublet, bs = broad singlet, bd = broad doublet, t = triplet, dd = doublet of doublets, and m = multiplet. High-resolution mass spectrometry (HRMS) is reported as the accumulation of 5 collections and was performed with an AB SCIEX 5800 matrix-assisted laser desorption ionization-time of flight/time of flight instrument in positive reflector mode with a delay time of 125 ns, mass tolerance of ±0.4 *m*/*z*, and α-cyano-4-hydroxycinnamic acid as the matrix and internal calibration.

#### (*S*)-1-(5-Methoxy-1,2,3,4-tetrahydronaphthalen-2-yl)piperazine (**2**)

(*S*)-5-Methoxy-1,2,3,4-tetrahydronaphthalen-2-amine HCl **1** (586 mg, 2.43 mmol, 1.00 equiv) was dissolved in 100 mL of EtOH in a 200 mL round-bottom flask. Bis(2-chloroethyl)amine HCl (867 mg, 4.68 mmol, 2.00 equiv) was added to the reaction followed by sodium bicarbonate (739 mg, 8.80 mmol, 3.60 equiv). The reaction was heated to reflux and stirred for 5.5 h. The reaction was cooled to room temperature and the solvent was evaporated in vacuo. The residue was resuspended in EtOAc (50 mL) along with water (3 mL). The aqueous layer was extracted with EtOAc (3 × 50 mL). The organic layers were combined and washed with saturated aqueous sodium bicarbonate (45 mL), followed by a wash of saturated aqueous sodium chloride (2 × 50 mL). The organic layer was dried over Na_2_SO_4_ to give 300 mg of a clear oil that was used later without further purification.

#### (*S*)-6-(Piperazin-1-yl)-5,6,7,8-tetrahydronaphthalen-1-ol (**3**)

The crude (*S*)-1-(5-methoxy-1,2,3,4-tetrahydronaphthalen-2-yl)piperazine **2** (88 mg, 0.360 mmol, 1.00 equiv) was suspended in 1.8 mL of 48% aq HBr (14.3 mmol, 40 equiv). The round-bottom flask was fitted with a reflux condenser and was stirred under reflux for 3.5 h. It was cooled to room temperature and the solvent was evaporated in vacuo to give 57 mg of a light tan solid that was used later without further purification.

#### (*S*)-6-(Piperazin-1-yl)-5,6,7,8-tetrahydronaphthalen-1-yl Trifluoromethanesulfonate (**4**)

The crude (*S*)-6-(piperazin-1-yl)-5,6,7,8-tetrahydronaphthalen-1-ol **3** (57 mg, 0.25 mmol, 1.00 equiv) was dissolved in 4 mL of anhydrous DCM in a dry 25 mL round-bottom flask. *N*-(2-Pyridyl)-bis(trifluoromethanesulfonimide) (96 mg, 0.37 mmol, 1.50 equiv) was added to the reaction at room temperature. The reaction was cooled to −78 °C using a dry ice-acetone bath and stirred for 5 min to allow the reaction to cool. *N*,*N*-Diisopropylethylamine (128 μL, 0.75 mmol, 3.00 equiv) was added dropwise at −78 °C. The reaction was warmed to room temperature and stirred for 20 h. Saturated aqueous ammonium chloride (15 mL) was used to quench the reaction on ice. The aqueous layer was extracted with DCM (3 × 15 mL). The organic layers were combined, dried over Na_2_SO_4_, filtered, and the solvent was evaporated in vacuo to give 91 mg of a light-yellow oil that was used below without further purification.

#### (*S*)-1-(5-(2-Fluorophenyl)-1,2,3,4-tetrahydronaphthalen-2-yl)piperazine (**FPIP**)

(*S*)-6-(Piperazin-1-yl)-5,6,7,8-tetrahydronaphthalen-1-yl trifluoromethanesulfonate **4** (91 mg, 0.26 mmol, 1.00 equiv) was dissolved in anhydrous 1,4-dioxane in a dry 25 mL round-bottom flask with a stir bar. 2-Fluorophenylboronic acid (146 mg, 1.04 mmol, 4.00 equiv) was added to the reaction. The solution was degassed with N_2_ for 30 min, and Pd(PPh_3_)_4_ (27 mg, 0.026 mmol, 0.10 equiv) was added along with KPO_3_ (83 mg, 0.39 mmol, 1.50 equiv) and KBr (35 mg, 0.29 mmol, 1.13 equiv). The reaction was heated to 120 °C under reflux for 6 h. The reaction was cooled, and the solvent was evaporated in vacuo. The reaction was resuspended using EtOAc (15 mL) and water (15 mL). The aqueous layer was extracted with EtOAc (2 × 15 mL). The organic fractions were combined and washed with saturated aqueous sodium chloride (2 × 15 mL) and dried over Na_2_SO_4_ and concentrated in vacuo. Purification by flash chromatography (5:1:0.1 Hex/EtOAc/TEA) gave 56 mg of a light-yellow oil. TLC solvents used were 5:5:1 Hex/EtOAc/TEA. The oil was converted to the corresponding HCl salt to yield a white solid (5 mg, 6.2%). ^1^H NMR (500 MHz; CDCl_3_): δ. 11.37 (s, 1H), 7.86 (d, *J* = 8.39 Hz, 1H), 7.64 (m, 1H), 7.46 (m, 1H), 7.32 (m, 1H), 7.05 (m, 2H), 6.73 (m, 1H), 4.05 (m, 2H), 3.92 (m, 2H), 3.65–3.56 (m, 2H), 3.11–3.02 (m, 1H), 2.94–2.87 (m, 1H), 2.85–2.80 (m, 1H), 2.26 (m, 1H), 1.66 (m, 1H). ^13^C NMR (500 MHz; CDCl_3_): δ 165.97, 134.31, 129.54, 127.90, 113.96, 67.78, 38.92, 30.58, 29.01, 24.00. ^19^F NMR (500 MHz; CDCl_3_): δ −114.82. HRMS calcd C_20_H_23_FN_2_ for [M + 2H]^+^, 312.1991; found, 312.1662.

#### (*S*)-5-Methoxy-*N*,*N*-dimethyl-1,2,3,4-tetrahydronaphthalen-2-amine (**5**)

(*S*)-5-Methoxy-1,2,3,4-tetrahydronaphthalen-2-amine HCl **1** (175 mg, 0.82 mmol, 1.00 equiv) was dissolved in 5 mL of MeOH in a 25 mL dry round-bottom flask, and formaldehyde (0.214 mL, 8.20 mmol, 10.0 equiv) was added. The reaction stirred at 130 °C under reflux for 2 h. The reaction was cooled on ice, and sodium borohydride (185 mg, 4.90 mmol, 6.00 equiv) was added slowly. The reaction was cooled to room temperature and stirred for 2 h. The solvent was evaporated in vacuo. The residue was resuspended with saturated ammonium chloride (25 mL) and extracted with DCM (3 × 25 mL). The organic layers were combined and dried over Na_2_SO_4_ to give 149 mg of a clear oil that was used later without further purification.

#### (*S*)-6-(Dimethylamino)-5,6,7,8-tetrahydronaphthalen-1-ol (**6**)

Crude (*S*)-5-methoxy-*N*,*N*-dimethyl-1,2,3,4-tetrahydronaphthalen-2-amine **5** (149 mg, 0.73 mmol, 1.00 equiv) was suspended in 3.6 mL of 48% aq HBr (29.0 mmol, 40 equiv). The round-bottom flask was fitted with a reflux condenser and stirred under reflux for 3.5 h. It was cooled to room temperature and the solvent was evaporated in vacuo to give 202 mg of a light tan solid.

#### (*S*)-6-(Dimethylamino)-5,6,7,8-tetrahydronaphthalen-1-yl Trifluoromethanesulfonate (**7**)

Crude (*S*)-6-(dimethylamino)-5,6,7,8-tetrahydronaphthalen-1-ol **6** (202 mg, 1.06 mmol, 1.00 equiv) was dissolved in 6 mL of anhydrous DCM in a dry 25 mL round-bottom flask. *N*-(2-Pyridyl)-bis(trifluoromethanesulfonimide) (451 mg, 1.58 mmol, 1.50 equiv) was added to the reaction at room temperature. The reaction was cooled to −78 °C using a dry ice-acetone bath and stirred for 5 min to allow the reaction to cool. *N*,*N*-Diisopropylethylamine (539 μL, 3.17 mmol, 3.00 equiv) was added dropwise at −78 °C. The reaction was warmed to room temperature and stirred for 20 h. Saturated aqueous ammonium chloride (25 mL) was used to quench the reaction on ice. The aqueous layer was extracted with DCM (3 × 25 mL). The organic layers were combined, dried over Na_2_SO_4_, filtered, and the solvent was evaporated in vacuo to give 60 mg of a light-yellow oil that was used later without further purification.

#### General Suzuki Coupling Conditions for Analogues **DiFPT** and **DBZ** (Scheme 1)

(*S*)-6-(Dimethylamino)-5,6,7,8-tetrahydronaphthalen-1-yl trifluoromethanesulfonate **7** was dissolved in anhydrous 1,4-dioxane in a dry 25 mL round-bottom flask with a stir bar. The corresponding boronic acid, 2,6-difluorophenylboronic acid (**DiFPT**) or 4-(dibenzofuranyl)boronic acid (**DBZ**) (4 equiv) was added to the reaction. The solution was degassed with N_2_ for 30 min and Pd(PPh_3_)_4_ (0.1 equiv) was added along with KPO_3_ (1.5 equiv) and KBr (1.13 equiv). The round-bottom flask was fitted with a reflux condenser and the reaction was heated to 120 °C under reflux for 6 h. The reaction was cooled, and the solvent was evaporated in vacuo. The reaction was resuspended using EtOAc and water. The aqueous layer was extracted with EtOAc (2×). The organic fractions were combined and washed with saturated aqueous sodium chloride and dried over Na_2_SO_4_ and concentrated in vacuo. Purification was done by flash chromatography (4:2:0.1 hexanes/EtOAc/TEA).

#### (*S*)-5-(2,6-Difluorophenyl)-*N*,*N*-dimethyl-1,2,3,4-tetrahydronaphthalen-2-amine (**DiFPT**)

The free base (2*S*)-**DiFPT** was obtained from **7** (0.19 mmol), as above, to give 54 mg of a clear oil. The oil was converted to the corresponding HCl salt to yield a white solid (24 mg, 75%). ^1^H NMR (500 MHz; CDCl_3_): δ 12.87 (s, 1H), 7.65 (m, 1H), 7.46 (m, 1H), 7.20 (m, 2H), 7.13 (d, *J* = 7.54 Hz, 1H), 7.09 (d, *J* = 8.07 Hz, 1H), 3.96–3.90 (m, 1H), 3.70–3.53 (m, 1H), 3.15 (d, *J* = 16.20 Hz, 2H), 2.49 (br s, 1H), 2.26 (m, 1H), 2.11 (s, 6H), 1.89 (bd, *J* = 6.41 Hz, 1H). ^13^C NMR (500 MHz; CDCl_3_): δ 165.95, 147.76, 135.29, 134.25, 129.48, 128.15, 127.77, 119.83, 117.28, 114.72, 67.78, 40.37, 38.96, 28.99, 23.99. ^19^F NMR (500 MHz; CDCl_3_): δ −114.57, −114.60. HRMS calcd C_18_H_19_F_2_N for [M + H]^+^, 288.1558; found, 288.1750.

#### (*S*)-5-(Dibenzo[*b*,*d*]furan-4-yl)-*N*,*N*-dimethyl-1,2,3,4-tetrahydronaphthalen-2-amine (**DBZ**)

The free base (2*S*)-**DBZ** was obtained from **7** (0.19 mmol), as above, to give 35 mg of a yellow oil. The oil was converted to the corresponding HCl salt to yield a white solid (31 mg, 49%). ^1^H NMR (500 MHz; CDCl_3_): δ 12.73 (s, 1H), 7.98 (dd, *J* = 7.59 Hz, 2H), 7.51 (d, *J* = 8.18 Hz, 1H), 7.46 (d, *J* = 7.10 Hz, 1H), 7.41–7.36 (m, 2H), 7.31 (d, *J* = 4.09 Hz, 1H), 7.28 (s, 1H), 7.27 (d, *J* = 6.06 Hz, 2H) 3.59 (s, 1H), 3.26 (dd, *J* = 14.37 Hz, 1H), 2.76 (s, 6H), 2.71 (t, *J* = 3.97 Hz, 1H), 2.33 (d, *J* = 10.95 Hz, 1H), 1.85 (s, 1H), 1.65 (s, 2H). ^13^C NMR (500 MHz; CDCl_3_): δ 156.43, 133.82, 132.49, 129.72, 128.80, 127.80, 127.02, 125.51, 124.66, 123.32, 121.13, 120.44, 112.24, 62.60, 39.87, 31.27, 30.40, 24.15. HRMS calcd C_24_H_23_NO for [M + H]^+^, 342.4615; found, 342.1953.

## Supporting Information Available

Representative results of dose–response binding of 5-SATs at 5-HT_1_ receptors; comparison of the 5-HT and 5-CT functional response at 5-HT_1_ receptors; molecular modeling of 5-CT at 5-HT_1_ receptors; comparison of basal and max functional response at mutated 5-HT_1_ receptors; comparison of 5-HT_1_ receptor binding pocket amino acids; affinity and functional potencies of lead compounds at all mutated 5-HT_1_ receptors; representative results of the functional response of 5-CT and 5-SATs at mutated 5-HT_1_ receptors; and conditions for PCR, binding assays, and functional assays at 5-HT_1_ receptors (PDF)

## Abbreviations

5-HT: 5-hydroxytryptamine
GPCR: G-protein coupled receptor
5-SAT: 5-substitued-2-aminotetralin
cAMP: cyclic adenosine monophosphate
5-CT: 5-carboxamidotryptamine
SAR: structure activity relationships
MD: molecular dynamic
CDCl_3_: deuterated chloroform
DCM: dichloromethane
EtOAc: ethyl acetate
EtOH: ethanol
HBr: hydrobromic acid
HCl: hydrochloric acid
Hex: hexanes
KBr: potassium bromide
KPO_3_: potassium phosphate
TEA: triethylamine
TLC: thin layer chromatography
MeOH: methanol
NMR: nuclear magnetic resonance
Na_2_SO_4_: sodium sulfate
Pd(PPh_3_)_4_: tetrakis(triphenylphosphine)palladium(0)
