# Supplementary material for: Development of 2-Aminotetralin-Type Serotonin 5-HT1 Agonists: Molecular Determinants for Selective Binding and Signaling at 5-HT1A, 5-HT1B, 5-HT1D, and 5-HT1F Receptors
Source: ACS Chem Neurosci. 2023 Dec 27;15(2):357–70. doi: 10.1021/acschemneuro.3c00658 (PMC10797628; doi:10.1021/acschemneuro.3c00658)
Supplement: Supplementary file 1 — cn3c00658_si_001.pdf [file cn3c00658_si_001.pdf]

Supporting Information

Development of 2-Aminotetralin-Type Serotonin 5-HT_1 agonists: Molecular Determinants for Selective Binding and Signaling at 5-HT_{1A}, 5-HT_{1B}, 5-HT_{1D}, and 5-HT_{1F} Receptors.

Ryan P. McGlynn[1,2,3*], Meng Cui[1,2], Brittany Brems[1,2,3], Otto Holbrook[1,2,3], Raymond G. Booth[1,2,3]

[1]Center for Drug Discovery, [2]Department of Pharmaceutical Sciences, and [3]Department of Chemistry and Chemical Biology, Northeastern University, Boston, MA, 02115

[*] Corresponding Author: mcglynn.r@northeastern.edu

Table of Contents

A. Supplemental Figures

Figure S1. Dose response curves of (S)-FPT at 5-$HT_{1A/1B/1D/1F}$ Receptors

Figure S2. Dose response Curves of 5-HT and 5-CT function at 5-HT_{1A} , 5-HT_{1B} , and 5-HT_{1D} receptors

Figure S3. Final binding poses of 5-CT at 5-HT_{1A}, 5-HT_{1B}, and 5-HT_{1D} receptors

Figure S4. Comparison of Basal and Max (10 µM 5-CT) Functional Response at 5-HT_{1A}, 5-HT_{1B} , and 5-HT_{1D} receptor constructs

Figure S5. 5-CT functional potency (pEC_{50}) at point mutated 5-HT_{1A}, 5-HT_{1B}, and 5-HT_{1D} receptors compared to wild type receptors.

Figure S6. Representative dose-response functional results of FPT at point mutated 5-HT_{1A}, 5-HT_{1B}, and 5-HT_{1D} receptors

B. Supplemental Tables

Table S1. Conserved and Non-Conserved Amino Acids at 5-HT_{1A}, 5-HT_{1B}, and 5-HT_{1D} Receptor Binding Pockets.

Table S2. Affinity (pKi) and Functional Potency (pEC_{50}) of FPT, PFPT, and NAP at Point Mutated 5-HT_{1A}, 5-HT_{1B}, and 5-HT_{1D} Receptors.

Table S3. Dissociation Constants and Protein Expression of 5-$HT_{1A/1B/1D}$ Point Mutated Receptors

Table S4. PCR conditions for Point Mutated 5-HT_{1A} Receptors

Table S5. PCR conditions for Point Mutated 5-HT_{1B} Receptors

Table S6. PCR conditions for Point Mutated 5-HT_{1D} Receptors

Table S7. Competitive Binding Assay Conditions for Wild Type and Point Mutated 5-HT_1 Subtype Receptors.

Table S8. Functional Assay Conditions for Wild Type and Point Mutated 5-HT_1 Subtype Receptors

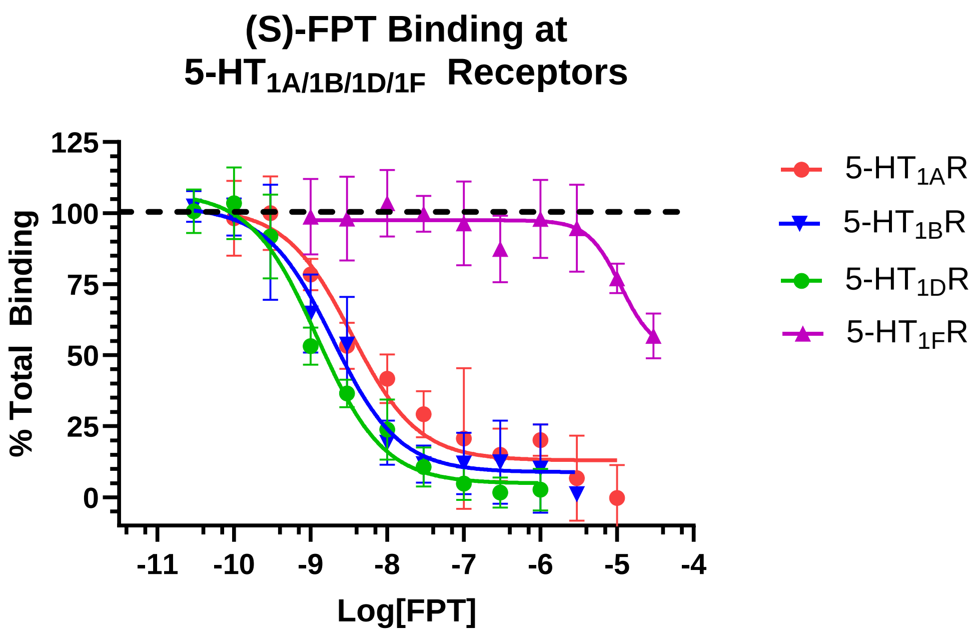

Figure S1. Dose response curves of (S)-FPT radioligand displacement at 5-$HT_{1A/1B/1D/1F}$ Receptors.

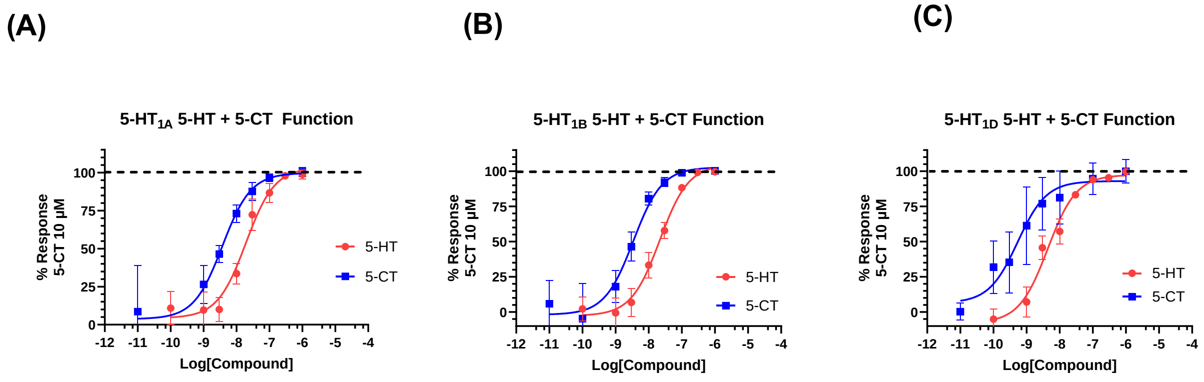

Figure S2. Dose response Curves of 5-HT and 5-CT function at 5-HT_{1A} (A), 5-HT_{1B} (B), and 5-HT_{1D} (C) receptors.

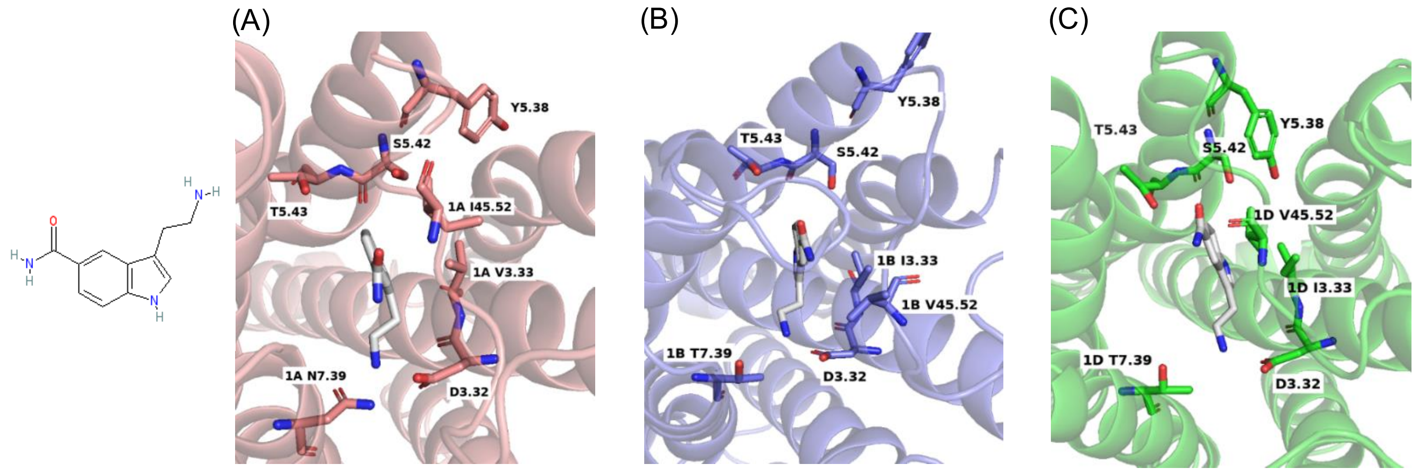

Figure S3. Final binding poses of 5-CT at 5-HT_{1A} (A), 5-HT_{1B} (B), and 5-HT_{1D} (C) receptors.

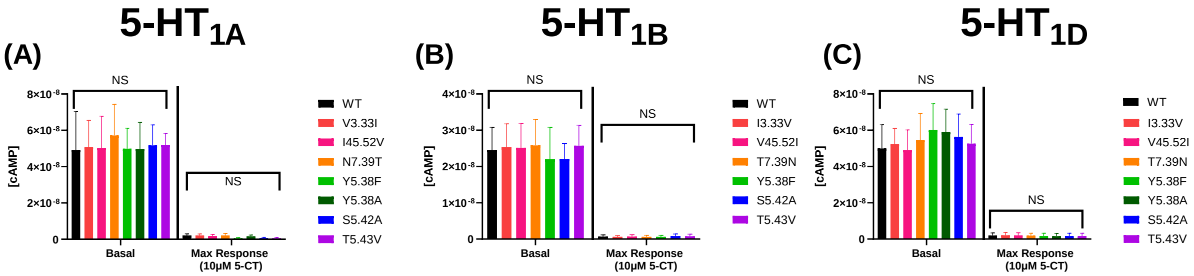

Figure S4. Comparison of Basal and Max Response (10 μM 5-CT) at 5-HT_{1A} (A), 5-HT_{1B} (B), and 5-HT_{1D} (C) receptor constructs

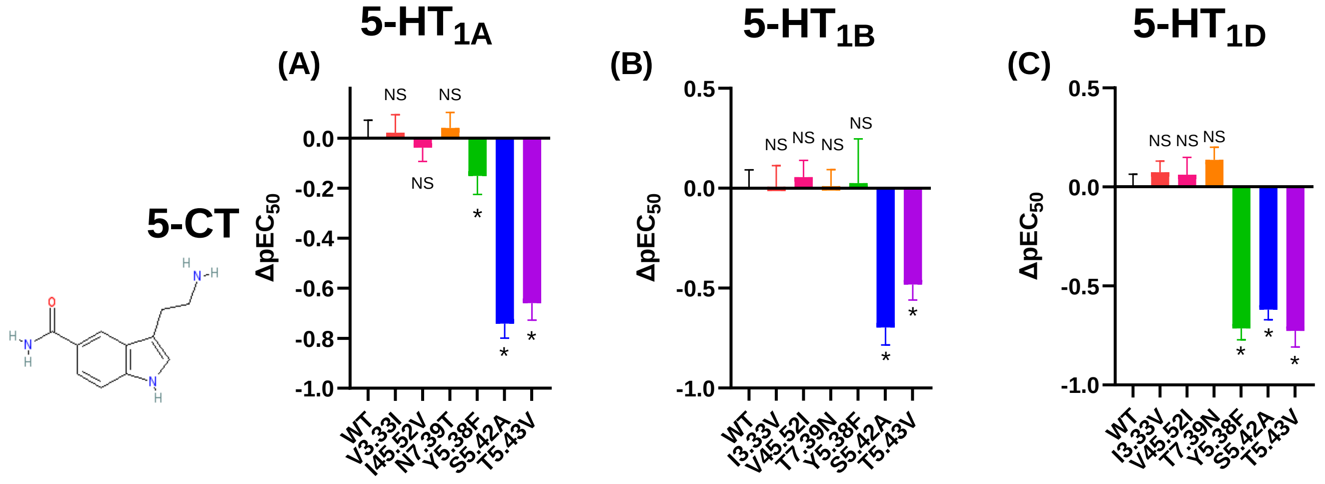

Figure S5. 5-CT functional potency (pEC_{50}) at point mutated 5-HT_{1A} (A), 5-HT_{1B} (B), and 5-HT_{1D} (C) receptors compared to wild type (WT) receptors. 'NS' denotes no significant change from wild type pEC_{50} determined by Student's t test, $P > 0.05$. (*) denotes a significant change from WT pEC_{50} determined by Student's t test, $P < 0.05$.

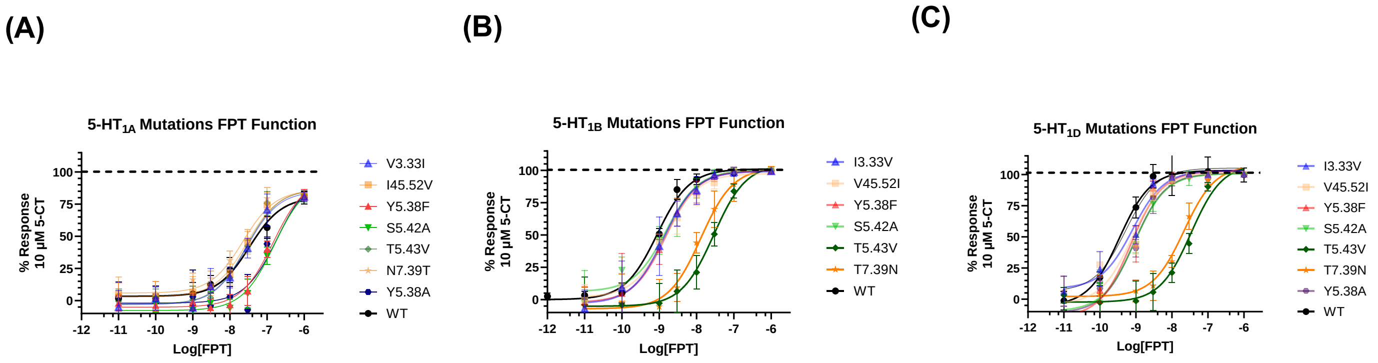

Figure S6. Representative dose-response functional results of FPT at point mutated 5-HT_{1A} (A), 5-HT_{1B} (B), and 5-HT_{1D} (C) receptors

Table S1. Conserved and Non-Conserved Amino Acids at 5-HT$_{1A}$, 5-HT$_{1B}$, and 5-HT$_{1D}$ Receptor Binding Pockets.

Position	5-HT$_{1A}$R	5-HT$_{1B}$R	5-HT$_{1D}$R
3.32	D	D	D
3.33	V	I	I
5.38	Y	Y	Y
5.42	S	S	S
5.43	T	T	T
45.52	I	V	V
7.39	N	T	T

Amino acid positions are displayed using Balleros Weinstein numbering. Positions that are non-conserved between 5-HT$_{1\ subtypes}$ are highlighted in yellow. Residues that are exclusively expressed in 5-HT$_{1A}$ receptors are highlighted in Blue. Residues that are exclusively expressed in 5-HT$_{1B}$ and 5-HT$_{1D}$ receptors are highlighted in Green.

Table S2. Affinity (pKi) and Functional Potency (pEC$_{50}$) of FPT, PFPT, and NAP at Point Mutated 5-HT$_{1A}$, 5-HT$_{1B}$, and 5-HT$_{1D}$ Receptors.

5-HT$_{1A}$ Receptor																
**Compound**	**WT**		**V3.33I**		**I45.52V**		**N7.39T**		**Y5.38F**		**Y5.38A**		**S5.42A**		**T5.43V**	
	pKi	pEC$_{50}$	pKi	pEC$_{50}$	pKi	pEC$_{50}$	pKi	pEC$_{50}$	pKi	pEC$_{50}$	pKi	pEC$_{50}$	pKi	pEC$_{50}$	pKi	pEC$_{50}$
**FPT**	8.39 ± 0.04	7.35 ± 0.12	8.46 ± 0.14	7.40 ± 0.04	8.36 ± 0.06	7.45 ± 0.02	*8.75 ± 0.08	*7.56± 0.06	*7.64 ± 0.12	*6.73 ± 0.08	*7.64 ± 0.14	*6.65 ± 0.04	*7.47 ± 0.07	*6.67 ± 0.08	8.34 ± 0.09	7.45 ± 0.07
**PFPT**	8.90 ± 0.21	8.78 ± 0.04	*8.09 ± 0.09	*7.98 ± 0.05	8.95 ± 0.10	8.87± 0.05	*8.07 ± 0.09	*7.60 ±0.06	8.76 ±0.12	8.68 ± 0.11	9.02 ± 0.18	8.69 ± 0.03	8.83± 0.12	8.79 ± 0.06	*7.90 ± 0.09	*7.61 ± 0.01
**NAP**	7.21 ± 0.14	6.41 ± 0.03	7.20 ± 0.06	6.45 ± 0.08	*8.20 ± 0.08	*7.44 ± 0.05	7.18 ±0.07	6.46 ± 0.03	7.22 ± 0.03	6.42 ± 0.06	*6.61 ± 0.04	*5.33 ± 0.07	7.20 ± 0.06	6.44 ± 0.02	7.19 ± 0.04	6.53 ± 0.08
**5-HT$_{1B}$ Receptor**																
	**WT**		**I3.33V**		**V45.52I**		**N7.39T**		**Y5.38F**		**S5.42A**		**T5.43V**			
	pKi	pEC$_{50}$	pKi	pEC$_{50}$	pKi	pEC$_{50}$	pKi	pEC$_{50}$	pKi	pEC$_{50}$	pKi	pEC$_{50}$	pKi	pEC$_{50}$		
**FPT**	8.65 ± 0.06	9.31 ± 0.08	8.57 ± 0.08	9.15 ± 0.08	8.72 ± 0.06	9.11 ± 0.08	*8.13 ± 0.03	*7.67 ±0.07	8.72 ± 0.06	9.09 ± 0.12	8.59 ± 0.10	8.99 ± 0.02	*8.01 ± 0.06	*7.46 ± 0.07		
**PFPT**	7.92 ± 0.10	7.25 ± 0.01	*8.26 ± 0.01	*7.72 ± 0.06	7.84 ± 0.08	7.29 ± 0.07	*8.23 ± 0.09	*7.71 ± 0.04	7.82 ± 0.08	7.26 ± 0.04	*6.99 ± 0.03	*6.50 ± 0.14	7.92 ± 0.02	7.28 ± 0.04		
**NAP**	8.34 ± 0.18	7.46 ± 0.07	8.28 ± 0.06	7.44 ± 0.05	*7.38 ± 0.04	*6.65 ± 0.04	8.19 ± 0.04	7.43 ± 0.03	8.25 ± 0.02	7.45 ± 0.03	8.23 ± 0.12	7.45 ± 0.03	8.21 ± 0.05	7.43 ± 0.08		
**5-HT$_{1D}$ Receptor**																
	**WT**		**I3.33V**		**V45.52I**		**N7.39T**		**Y5.38F**		**Y5.38A**		**S5.42A**		**T5.43V**	
	pKi	pEC$_{50}$	pKi	pEC$_{50}$	pKi	pEC$_{50}$	pKi	pEC$_{50}$	pKi	pEC$_{50}$	pKi	pEC$_{50}$	pKi	pEC$_{50}$	pKi	pEC$_{50}$
**FPT**	8.97 ± 0.20	8.64 ± 0.61	8.94 ± 0.02	8.86 ± 0.04	8.98 ± 0.08	8.83 ± 0.15	*7.97 ± 0.08	*7.85 ± 0.06	8.89 ± 0.03	8.81 ± 0.06	8.97 ± 0.12	8.84 ± 0.18	8.89 ± 0.15	8.87 ± 0.19	*7.85 ± 0.07	*7.52 ± 0.11
**PFPT**	7.87 ± 0.10	7.24 ± 0.05	*8.44 ± 0.05	*7.64 ± 0.03	7.83 ± 0.12	7.23 ± 0.02	*8.38 ± 0.13	*7.73 ± 0.01	*7.38 ± 0.08	*6.62 ± 0.08	*7.37 ± 0.04	*6.72 ± 0.12	*7.38 ± 0.09	*6.51 ± 0.12	7.89 ± 0.06	7.28 ± 0.05
**NAP**	9.08 ± 0.21	8.06 ± 0.22	8.95 ± 0.08	8.03 ± 0.09	*7.43 ± 0.08	*6.59 ± 0.14	8.97 ± 0.17	8.00 ± 0.08	9.18 ± 0.30	7.95 ± 0.08	*7.15 ± 0.02	*6.54 ± 0.01	8.93 ± 0.13	8.00 ± 0.13	9.05 ± 0.1	7.98 ± 0.02

Results are displayed as mean ± SD derived from 3 independent experiments. (*) Denotes a significantly difference from wild type receptor as determined by Student's t test, $P < 0.05$

Table S3. Dissociation Constants and Protein Expression of 5-$HT_{1A/1B/1D}$ Point Mutated Receptors.

Parameter	5-HT_{1A} receptor Variants							
	WT	V3.33I	I45.52V	N7.39T	Y5.38F	Y5.38A	S5.42A	T5.43V
K_D (nM)	2.5 ± 0.2	2.5 ± 0.3	2.5 ± 0.4	2.7 ± 0.1	*5.5 ± 0 .2	*6.0 ± 0.1	*13.2 ± 0.1	*10.8 ± 0.8
B_{MAX} (pmol/mg protein)	3.8 ± 0.4	2.9 ± 0.5	3.2 ± 0.4	3.3 ± 0.3	3.1 ± 0.6	3.0 ± 0.1	3.8 ± 0.4	3.7 ± 0.2
	5-HT_{1B} Receptor Variants							
	WT	I3.33V	V45.52I	T7.39N	S5.42A	T5.43V		
K_D (nM)	5.3±0.5	5.4 ± 0.2	5.9 ± 0.4	5.6 ± 0.3	*11.8 ± 0.6	*9.4 ± 0.1		
B_{MAX} (pmol/mg protein)	3.2 ± 0.6	3.1 ± 0.4	3.0 ± 0.3	3.2 ± 0.08	3.6 ± 0.3	3.4 ± 0.5		
	5-HT_{1D} Receptor Variants							
	WT	I3.33V	V45.52I	T7.39N	Y5.38F	Y5.38A	S5.42A	T5.43V
K_D (nM)	0.82 ± 0.1	0.75 ± 0.1	0.95 ± 0.02	0.87 ± 0.2	*3.3±0.2	*6.2 ± 0.02	*7.6 ± 0.2	*7.98 ± 0.2
B_{MAX} (pmol/mg protein)	1.6 ± 0.4	1.2 ± 0.2	2.0 ± 0.1	1.3 ± 0.1	1.5 ± 0.3	2.1 ± 0.4	1.6 ± 0.07	1.8 ± 0.4

Table S4. PCR conditions for Point Mutated 5-HT_{1A} Receptors

A	5-HT_{1A}						
B	V3.33I	I45.52V	N7.39T	Y5.38F	Y5.38A	S5.42A	T5.43V
C	5'-atgaggtgcagcacagtatgtcgagggcgatgaac-3'	5'-catgatccttgctaacggtgcatgcgtcggg-3'	5’-gtagccgacgtaagtgattatcgcgcccaag-3’	5'-caaaggtggaatagatagtgaagccatgatccttgctaatg-3'	5'-caaaggtggaatagatagtggcgccatgatccttgctaatg-3	5'-ctccaaaggtggcatagatagtgtagccatgatcct-3'	5'-gggatgtagaaagctccaaagacggaatagatagtgtagccatg-3'
D	5'-gttcatcgccctcgacatactgtgctgcacctcat-3'	5'-cccgacgcatgcaccgttagcaaggatcatg-3'	5'-cttgggcgcgataatcacttacctgggctac-3'	5'-cattagcaaggatcatggcttcactatctattccacctttg-3'	5'-cattagcaaggatcatggccgcactatctattccacctttg-3'	5'-aggatcatggctacactatctatgccaccttggag-3'	5'-catggctacactatctattccgtctttggagctttctacatccc-3'
E	95ºC						
F	95ºC						
G	65ºC	64ºC	62ºC	70ºC	70ºC	67ºC	69ºC
H	72ºC						
I	72ºC						
J	4ºC						
K	25						
L	10						

A = Receptor Subtype. B = Point Mutation. C = FW Primer. D = REV primer. E = Initial denaturation (60s). F = denaturation (60s). G = annealing (60s). H = Extension (10min). I = Final extension. J = Hold (O/N). K = # of cycles. L = Quantity of template DNA (ng)

Table S5. PCR conditions for Point Mutated 5-HT$_{1B}$ Receptors

A	5-HT$_{1B}$					
B	I3.33V	V45.52I	T7.39N	Y5.38F	S5.42A	T5.43V
C	5'-cagtgcaacaagtgacgtccgacgacagcca-3'	5'-gtgactgaatgcgtgatcaacaccgaccac-3'	5'-cctttgacttcttcaattggctgggctat-3'	5'-accagccagattagctttaccatttataccatt-3"	5'-atttataccatttatgccacctgcggcgcgttt-3'	5'-tataccatttatagcgtctgcggcgcgttttat-3'
D	5'-tggctgtcgtcggacgtcacttgttgcactg-3'	5'-gtggtcggtgttgatcacgcattcagtcac-3'	5'-atagcccagccaattgaagaagtcaaagg-3'	5'-aatggtataaatggtaaagctaatctggctggt -3'	5'-aaacgcgccgcaggtggcataaatggtataaat-3'	5'-ataaaacgcgccgcagacgctataaatggtata-3'
E	95ºC					
F	95ºC					
G	62ºC	62ºC	62ºC	68ºC	62ºC	64ºC
H	72ºC					
I	72ºC					
J	4ºC					
K	25					
L	10					

A = receptor subtype. B = Point Mutation. C = FW Primer. D = REV primer. E = Initial denaturation (60s). F = denaturation (60s). G = annealing (60s). H = Extension (10min). I = Final extension. J = Hold (O/N). K = # of cycles. L = Quantity of template DNA (ng)

Table S6. PCR conditions for Point Mutated 5-HT_{1D} Receptors

A	5-HT_{1D}						
B	I3.33V	V45.52I	T7.39N	Y5.38F	Y5.38A	S5.42A	T5.43V
C	5'-cagtgcaacaagtgacgtccgacgacagcca-3'	5'-gtgactgaatgcgtgatcaacaccgaccac-3'	5'-cctttgacttcttcaattggctgggctat-3'	5’-accagccagattagctttaccatttataccatt-3”	5'-caaaggtggaatagatagtggcgccatgatccttgctaatg-3'	5’-atttataccatttatgccacctgcggcgcgttt-3’	5’-tataccatttatagcgtctgcggcgcgttttat-3’
D	5'-tggctgtcgtcggacgtcacttgttgcactg-3'	5'-gtggtcggtgttgatcacgcattcagtcac-3'	5'-atagcccagccaattgaagaagtcaaagg-3'	5’-aatggtataaatggtaaagctaatctggctggt -3’	5'-cattagcaaggatcatggccgcactatctattccaccttg-3'	5’-aaacgcgccgcaggtggcataaatggtataaat-3’	5’-ataaaacgcgccgcagacgctataaatggtata-3’
E	95ºC						
F	95ºC						
G	62ºC	62ºC	62ºC	68ºC	72ºC	62ºC	64ºC
H	72ºC						
I	72ºC						
J	4ºC						
K	25						
L	10						

A = Receptor Subtype. B = Point Mutation. C = Forward Primer. D = Reverse primer. E = Initial denaturation (60s). F = denaturation (60s). G = annealing (60s). H = Extension (10min). I = Final extension. J = Hold (O/N). K = # of cycles. L = Quantity of template DNA (ng)

Table S7. Competitive Binding Assay Conditions for Wild Type and Point Mutated 5-HT$_1$ Subtype Receptors.

Receptor	n	Radioligand	Non-Specific Binding (10 μM)	Incubation (min)	Buffer	K_d (nM ± SD)	B_{MAX} (pmol/mg ±SD)
5-HT$_{1A}$ WT	3	[^{3}H]5-CT	5-HT	90	STD	2.5 ± 0.2	3.8 ± 0.5
5-HT$_{1B}$ WT	3	[^{3}H]5-CT	5-HT	90	STD	5.34 ± 0.46	3.2 ± 0.6
5-HT$_{1D}$ WT	3	[^{3}H]5-CT	5-HT	90	STD	0.82 ± 0.1	1.6 ± 0.4
5-HT$_{1F}$ WT	3	[^{3}H]5-HT	LY334730	90	STD	7.8 ± 2.3	8.3 ± 0.5
5-HT$_{1A}$ V3.33I	2	[^{3}H]5-CT	5-HT	90	STD	2.5 ± 0.3	2.9 ± 0.5
5-HT$_{1A}$ I45.52V	2	[^{3}H]5-CT	5-HT	90	STD	2.5 ± 0.4	3.2 ± 0.4
5-HT$_{1A}$ N7.39T	2	[^{3}H]5-CT	5-HT	90	STD	2.7 ± 0.01	3.3 ± 0.3
5-HT$_{1A}$ Y5.38F	2	[^{3}H]5-CT	5-HT	90	STD	5.5 ± 0.2	3.1 ± 0.6
5-HT$_{1A}$ Y5.38A	2	[^{3}H]5-CT	5-HT	90	STD	6.0 ± 0.1	3.0 ± 0.1
5-HT$_{1A}$ S5.42A	2	[^{3}H]5-CT	5-HT	90	STD	13.2 ± 0.1	3.8 ± 0.4
5-HT$_{1A}$ T5.43V	2	[^{3}H]5-CT	5-HT	90	STD	10.8 ± 0.8	3.7 ± 0.2
5-HT$_{1B}$ I3.33V	2	[^{3}H]5-CT	5-HT	90	STD	5.4 ± 0.2	3.1 ± 0.4
5-HT$_{1B}$ V45.52I	2	[^{3}H]5-CT	5-HT	90	STD	5.9 ± 0.4	3.0 ± 0.3
5-HT$_{1B}$ T7.39N	2	[^{3}H]5-CT	5-HT	90	STD	5.6 ± 0.3	3.2 ± 0.1
5-HT$_{1B}$ S5.42A	2	[^{3}H]5-CT	5-HT	90	STD	11.8 ± 0.6	3.6 ± 0.3
5-HT$_{1B}$ T5.43V	2	[^{3}H]5-CT	5-HT	90	STD	9.4 ± 0.1	3.4 ± 0.5
5-HT$_{1D}$ I3.33V	2	[^{3}H]5-CT	5-HT	90	STD	0.75 ± 0.1	1.2 ± 0.2
5-HT$_{1D}$ V45.52I	2	[^{3}H]5-CT	5-HT	90	STD	0.95 ± 0.1	2.0 ± 0.1
5-HT$_{1D}$ T7.3	2	[^{3}H]5-CT	5-HT	90	STD	0.87 ± 0.2	1.3 ± 0.1
5-HT$_{1D}$ Y5.38F	2	[^{3}H]5-CT	5-HT	90	STD	3.3 ± 0.2	1.5 ± 0.3
5-HT$_{1D}$ Y5.38A	2	[^{3}H]5-CT	5-HT	90	STD	6.2 ± 0.1	2.1 ± 0.4
5-HT$_{1D}$ S5.42A	2	[^{3}H]5-CT	5-HT	90	STD	7.6 ± 0.2	1.6 ± 0.7
5-HT$_{1D}$ T5.43V	2	[^{3}H]5-CT	5-HT	90	STD	8.0 ± 0.2	1.8 ± 0.4

Summary of radioligand competition binding assays at various 5-HT$_1$ receptor constructs. STD is 50 mM Tris HCl, 10 mM $MgCl_2$ and 0.1 mM EDTA in filtered deionized water.

Table S8. Functional Assay Conditions for Wild Type and Point Mutated 5-HT$_1$ Subtype Receptors

Cell Type	Receptor	Cell Density	[Forskolin]	Incubation time (37°C)
HEK293$_t$	5-HT$_{1A}$ WT	300 cells/μL	300 nM	90 min
HEK293$_t$	5-HT$_{1B}$ WT	500 cells/μL	600 nM	90 min
HEK293$_t$	5-HT$_{1D}$ WT	250 cells/μL	300 nM	90 min
HEK293$_t$	5-HT$_{1A}$ V3.33I	300 cells/μL	300 nM	90 min
HEK293$_t$	5-HT$_{1A}$ I45.52V	300 cells/μL	300 nM	90 min
HEK293$_t$	5-HT$_{1A}$ N7.39T	300 cells/μL	300 nM	90 min
HEK293$_t$	5-HT$_{1A}$ Y5.38F	300 cells/μL	300 nM	90 min
HEK293$_t$	5-HT$_{1A}$ Y5.38A	300 cells/μL	300 nM	90 min
HEK293$_t$	5-HT$_{1A}$ S5.42A	300 cells/μL	300 nM	90 min
HEK293$_t$	5-HT$_{1A}$ T5.43V	300 cells/μL	300 nM	90 min
HEK293$_t$	5-HT$_{1B}$ I3.33V	500 cells/μL	600 nM	90 min
HEK293$_t$	5-HT$_{1B}$ V45.52I	500 cells/μL	600 nM	90 min
HEK293$_t$	5-HT$_{1B}$ N7.39T	500 cells/μL	600 nM	90 min
HEK293$_t$	5-HT$_{1B}$ Y5.38F	500 cells/μL	600 nM	90 min
HEK293$_t$	5-HT$_{1B}$ S5.42A	500 cells/μL	600 nM	90 min
HEK293$_t$	5-HT$_{1B}$ T5.43V	250 cells/μL	600 nM	90 min
HEK293$_t$	5-HT$_{1D}$ I3.33V	250 cells/μL	300 nM	90 min
HEK293$_t$	5-HT$_{1D}$ V45.52I	250 cells/μL	300 nM	90 min
HEK293$_t$	5-HT$_{1D}$ N7.39T	250 cells/μL	300 nM	90 min
HEK293$_t$	5-HT$_{1D}$ Y5.38F	250 cells/μL	300 nM	90 min
HEK293$_t$	5-HT$_{1D}$ Y5.38A	250 cells/μL	300 nM	90 min
HEK293$_t$	5-HT$_{1D}$ S5.42A	250 cells/μL	300 nM	90 min
HEK293$_t$	5-HT$_{1D}$ T5.43V	250 cells/μL	300 nM	90 min

Summary of functional assay conditions at various 5-HT$_1$ receptor point mutation constructs.
